# Biochemical Remodeling of Purine Metabolism and Lipid Peroxidation Across Thyroid Disease Phenotypes

**DOI:** 10.3390/metabo16100700

**Published:** 2026-09-22

**Authors:** Ernur Bekov, Olga Ponamareva, Ryszhan Bakirova, Vilen Molotov-Luchanskiy, Nazgul Omarbekova, Khamida Abdikadirova, Azamat Kharin, Yelena Pozdnyakova

**Affiliations:** 1 Department of Internal Medicine, Karaganda Medical University, Karaganda 100008, Kazakhstan; 2 Department of Biomedicine, Karaganda Medical University, Karaganda 100008, Kazakhstan; 3 Department of Informatics and Biostatistics, Karaganda Medical University, Karaganda 100008, Kazakhstan; 4 Department of Physiology, Karaganda Medical University, Karaganda 100008, Kazakhstan

**Keywords:** autoimmune thyroiditis, hypothyroidism, thyroid dysfunction, purine catabolism, xanthine oxidoreductase, hypoxanthine, uric acid, malondialdehyde, erythrocytes, oxidative stress

## Abstract

**Highlights:**

**What are the main findings?**

- Thyroid disease phenotypes show distinct, compartment-specific purine-metabolite patterns in plasma and erythrocytes rather than a uniform AIT-dependent metabolic shift.
- Plasma TBARS-derived MDA equivalents were increased across all thyroid-disease groups, whereas erythrocyte TBARS-derived MDA equivalents showed a more selective phenotype-specific pattern.

**What are the implications of the main findings?**

- The combined purine-metabolite and MDA findings highlight a previously underexplored relationship between altered purine profiles and lipid peroxidation across thyroid disease phenotypes.
- With prospective validation, combined purine-metabolite and lipid-peroxidation profiles may provide a basis for multiparametric biomarker panels for biochemical stratification and longitudinal monitoring of thyroid disorders.

**Abstract:**

**Background and Objectives**: Purine-metabolism profiles across distinct thyroid phenotypes remain poorly characterized despite the recognized redox imbalance associated with thyroid dysfunction and autoimmune thyroiditis (AIT). We compared plasma and erythrocyte purine-metabolite profiles, derived purine ratios, and their associations with lipid peroxidation across four thyroid-disease phenotypes and healthy controls. **Materials and Methods**: This single-center cross-sectional study included 164 participants: hypothyroidism without AIT (*n* = 31), AIT with hypothyroidism (*n* = 30), nontoxic nodular goiter with euthyroidism (*n* = 42), euthyroid AIT (*n* = 37), and healthy controls (*n* = 24). Purine metabolites were assessed by direct UV spectrophotometry and expressed in relative extinction units; thiobarbituric acid-reactive substances (TBARS)-derived malondialdehyde (MDA) equivalents were measured in plasma and erythrocytes. **Results**: Plasma guanine and hypoxanthine were lower in AIT with hypothyroidism than in hypothyroidism without AIT and in euthyroid AIT than in nontoxic nodular goiter. Plasma X/HX and UA/HX were higher in both AIT groups than in controls. Plasma MDA was elevated in all thyroid-disease groups, whereas erythrocyte MDA was highest in hypothyroidism without AIT. Plasma UA/X correlated positively with both plasma and erythrocyte MDA, and plasma UA/HX with plasma MDA, with phenotype-dependent heterogeneity in stratified analyses. **Conclusions**: Thyroid phenotypes showed distinct compartment-specific purine and lipid-peroxidation patterns rather than a uniform AIT-dependent shift. Purine ratios should be interpreted as indirect, method-specific indices rather than direct measures of metabolic flux or XOR/XO/XDH activity.

## 1. Introduction

Autoimmune thyroiditis (AIT), or Hashimoto’s thyroiditis, is one of the most common autoimmune diseases of the thyroid gland and one of the leading causes of hypothyroidism in iodine-sufficient regions [1,2,3]. AIT occurs significantly more often in women, especially in the 30–50-year age group; according to recent reviews, the prevalence of Hashimoto’s thyroiditis in women varies depending on the region, diagnostic criteria, and study population and may reach 17–25% in selected cohorts [4,5]. AIT is one of the most frequent reasons for referral to an endocrinologist among patients with thyroid pathology; the disease may occur as an isolated condition or in combination with thyroid dysfunction, primarily subclinical or overt hypothyroidism [6,7].

Excessive iodine intake is an environmental factor associated with thyroid autoimmunity in susceptible individuals and has been linked to increased thyroglobulin (TG) immunogenicity, enhanced oxidative stress in thyrocytes, local inflammatory activation, and increased thyroid autoantibody positivity [8,9,10,11]. Redox imbalance is mechanistically relevant because thyroid hormone synthesis requires tightly regulated hydrogen peroxide (H_2_O_2_) generation for thyroid peroxidase (TPO)-mediated iodide oxidation and TG iodination; excessive H_2_O_2_ production or insufficient antioxidant neutralization may increase lipid peroxidation, promote thyrocyte injury, and contribute to loss of immune tolerance to thyroid autoantigens [12,13,14,15,16,17].

The relationship between thyroid-hormone status, free-radical oxidation processes –including lipid peroxidation and oxidative modification of proteins and carbohydrates –and purine metabolism remains insufficiently characterized. Purine catabolism at the stage of hypoxanthine (HX) conversion to xanthine (X) and subsequently to uric acid (UA) is catalyzed by xanthine oxidoreductase (XOR), which exists in two interconvertible forms: xanthine dehydrogenase (XDH), which uses nicotinamide adenine dinucleotide (NAD^+^) as an electron acceptor, and xanthine oxidase (XO), which interacts with molecular oxygen. A shift in XOR activity toward XO is accompanied by increased generation of reactive oxygen species (ROS), including superoxide anion and H_2_O_2_, thereby enhancing the pro-oxidant burden [18,19]. Malondialdehyde (MDA), despite its limited analytical specificity, is widely used in clinical studies as an integral marker of lipid peroxidation [20,21]. Joint assessment of purine-metabolite parameters and MDA may provide complementary information on the relationship between purine metabolism and lipid peroxidation. Because purine-metabolite distributions may be influenced by multiple metabolic processes, such associations should not be interpreted as direct evidence of increased XOR activity, XOR-derived ROS generation, or metabolic flux [22,23].

In hypothyroidism, reduced thyroid-hormone signaling is associated with impaired energy metabolism, including decreased mitochondrial biogenesis, respiratory-chain activity, oxidative phosphorylation, and adenosine triphosphate (ATP) production [24,25].

In AIT, oxidative stress is additionally linked to immune dysfunction and disturbances of lipid peroxidation [26,27].

Despite available data on the association between thyroid dysfunction and uric acid levels, systematic studies of the purine metabolite profile in thyroid pathology remain limited. Most studies focus primarily on the final product of purine catabolism, uric acid, whereas intermediate metabolites, including hypoxanthine and xanthine, as well as their ratios, are assessed much less frequently [28,29]. An integrated analysis of purine-metabolite profiles and MDA may help characterize differences in the relative distribution of purine metabolites and their associations with lipid-peroxidation markers across thyroid phenotypes. This may be relevant to mechanistic understanding and to the future development of markers for biochemical stratification and monitoring.

The aim of this study was to characterize the profile of intermediate purine catabolites and the X/HX, UA/X, and UA/HX relative purine-metabolite indices in plasma and erythrocytes in patients with different thyroid-disease phenotypes (with/without AIT; hypo-/euthyroidism) and to evaluate their association with MDA as a marker of lipid peroxidation.

## 2. Materials and Methods

### 2.1. Study Design

This was a single-center, observational, cross-sectional study. The study was conducted at the shared-use laboratory and the laboratory of the Department of Biological Chemistry of the Non-Commercial Joint-Stock Company “Karaganda Medical University” (Karaganda, Kazakhstan) from 2022 to 2024.

During the study period, 172 individuals were assessed for eligibility. Of these, 8 individuals were excluded because they did not meet the predefined eligibility criteria, and 164 participants were included in the final analysis. No repeat sample collection was required because of pre-analytical quality-control criteria.

A total of 164 participants were included in the study. Participants were assigned to five predefined groups according to clinical thyroid diagnosis, thyroid functional status, thyroid autoantibody status, and, where applicable, ultrasonographic evidence of nodular thyroid disease:

Group 1—patients with hypothyroidism without AIT (Hypo without AIT; *n* = 31): participants with subclinical or overt hypothyroidism and negative thyroid autoantibody status, defined as anti-thyroid peroxidase antibody (anti-TPO) concentrations < 35 IU/mL and anti-thyroglobulin antibody (anti-TG) concentrations < 115 IU/mL. None of the participants in Group 1 had a history of thyroid surgery or radioiodine treatment. No further etiological subclassification was performed within this group.

Group 2—AIT with hypothyroidism (AIT + Hypo; *n* = 30): participants with an endocrinologist-established diagnosis of AIT, subclinical or overt hypothyroidism, and thyroid autoantibody positivity defined as anti-TPO ≥ 35 IU/mL and/or anti-TG ≥ 115 IU/mL.

Group 3—patients with nontoxic nodular goiter with euthyroidism (NNG, euthyroid; *n* = 42): participants with thyroid nodules identified by ultrasonography, euthyroid thyroid function, and negative thyroid autoantibody status. Euthyroidism was defined as a thyroid-stimulating hormone (TSH) concentration of 0.40–4.00 mIU/L and a free thyroxine (fT4) concentration of 10–22 pmol/L; anti-TPO and anti-TG concentrations were below the predefined seropositivity thresholds.

Group 4—patients with euthyroid AIT (Euthyroid AIT; *n* = 37): participants with an endocrinologist-established diagnosis of AIT, thyroid autoantibody positivity defined as anti-TPO ≥ 35 IU/mL and/or anti-TG ≥ 115 IU/mL, and preserved thyroid function, defined as TSH 0.40–4.00 mIU/L and fT4 10–22 pmol/L.

Group 5—healthy controls (Controls; *n* = 24): clinically healthy participants with no diagnosed thyroid disease, euthyroid thyroid function, and negative thyroid autoantibody status, defined as anti-TPO < 35 IU/mL and anti-TG < 115 IU/mL.

Participants were assigned to the study groups using the same predefined classification criteria throughout the study. The primary analysis was designed to compare laboratory parameters across these five clinical phenotypes. Sex- and age-stratified analyses were not performed because of the limited sample sizes within the individual groups. All participants were within the predefined age range of 18–59 years.

### 2.2. Operational Definitions

Euthyroidism was defined as a TSH concentration of 0.40–4.00 mIU/L in combination with an fT4 concentration of 10–22 pmol/L.

Subclinical hypothyroidism was defined as a TSH concentration > 4.00 mIU/L with an fT4 concentration within the reference range.

Overt hypothyroidism was defined as a TSH concentration > 4.00 mIU/L accompanied by an fT4 concentration < 10 pmol/L.

For the purposes of study-group classification, thyroid autoantibody positivity was operationally defined as an anti-TPO concentration ≥ 35 IU/mL and/or an anti-TG concentration ≥ 115 IU/mL, whereas values below both thresholds were classified as antibody-negative. For Groups 2 and 4, assignment to the AIT phenotype was based on an endocrinologist-established clinical diagnosis together with fulfillment of the predefined antibody criterion. Ultrasonographic features compatible with chronic autoimmune thyroid involvement, when available, were considered supportive evidence. These antibody thresholds were used as predefined operational criteria for cohort classification and should not be interpreted as universal diagnostic cutoffs, since antibody titers may vary with age, disease stage, and individual clinical context.

Nontoxic nodular goiter was defined by the presence of one or more thyroid nodules identified by ultrasonography in participants with preserved thyroid function and negative thyroid autoantibody status. Renal function was characterized using the estimated glomerular filtration rate (eGFR), calculated according to the CKD-EPI 2021 equation and expressed as mL/min/1.73 m^2^. eGFR was included in the baseline clinical characterization because renal filtration is an important determinant of circulating uric acid and may also influence the distribution of other purine metabolites.

In participants receiving levothyroxine, venous blood was collected in the morning before the next scheduled dose. Information on levothyroxine use, daily dose, body-weight-adjusted dose (µg/kg/day), and treatment duration was recorded at enrollment.

### 2.3. Inclusion and Exclusion Criteria

The inclusion criteria were as follows: age 18–59 years; availability of TSH, fT4, anti-TPO, and anti-TG results; an endocrinologist’s clinical assessment sufficient to assign the participant to one of the predefined study groups; availability of the clinical and, where applicable, ultrasonographic information required for phenotype classification; availability of blood samples suitable for the determination of purine-metabolism parameters and thiobarbituric acid-reactive substances (TBARS)-derived MDA in plasma and the erythrocyte fraction; and written informed consent to participate in the study.

The exclusion criteria were as follows: age < 18 or >59 years; pregnancy or lactation; acute infectious or inflammatory disease within 4 weeks before blood collection; exacerbation of a chronic medical condition; concomitant endocrine disorders, including thyrotoxicosis, subacute thyroiditis, type 1 or type 2 diabetes mellitus, and other clinically significant endocrine disorders; severe cardiovascular, hepatic, or renal failure; active malignancy; clinically significant hematologic disorders; and the use of medications or dietary supplements that met the predefined medication-related exclusion criteria because of their potential to materially affect purine-metabolism or oxidative-stress measurements.

High-sensitivity C-reactive protein (hs-CRP), erythrocyte sedimentation rate (ESR), and hemogram-derived systemic inflammatory indices, including the neutrophil-to-lymphocyte ratio (NLR), were not prespecified study variables and were not systematically available across the cohort. The exclusion of participants with acute infectious or inflammatory disease minimized the influence of overt acute inflammation; however, chronic low-grade systemic inflammatory activity was not directly quantified.

Medication and dietary supplement use during the preceding 4 weeks was recorded for all participants using a standardized questionnaire. Participants receiving antioxidant preparations, including vitamins C and E or N-acetyl-L-cysteine, or other medications or supplements meeting the predefined medication-related exclusion criteria were excluded.

Biological samples were excluded from laboratory analysis in cases of marked hemolysis, lipemia, or icterus; repeated freeze–thaw cycles; or noncompliance with predefined preanalytical storage and transportation requirements.

### 2.4. Ethical Considerations

The study was conducted in accordance with the principles of the Declaration of Helsinki. Written informed consent was obtained from all participants. The study protocol was approved by the Ethics Committee of Karaganda Medical University (Protocol No. 1 dated 18 September 2022).

### 2.5. Biological Material and Pre-Analytical Processing

Plasma obtained from heparinized blood and the erythrocyte fraction were used for the determination of purine metabolites, whereas serum was used for assessment of the hormonal profile. Venous blood was collected in the morning after an overnight fast of 8–12 h.

For plasma preparation, blood was collected into 10 mL vacuum tubes containing lithium heparin (green cap; UNIVAC™/Eiliton LLC, Dubna, Russia). Blood samples were centrifuged at 2000× *g* for 10 min at 4 °C. Plasma was separated and aliquoted within 30 min after blood collection. For short-term storage, plasma samples were maintained at +2 to +8 °C for no longer than 48 h; for long-term storage, plasma aliquots were stored at −80 °C. Repeated freeze–thaw cycles were not allowed.

Serum for ELISA was obtained from whole blood collected into 5 mL tubes containing a clot activator and separating gel (UNIVAC™/Eiliton LLC, Dubna, Russia). Samples were kept upright for 30–45 min until complete clot formation and then centrifuged at 1500× *g* for 10 min at 20–22 °C. Serum was transferred into clean polypropylene tubes and stored at +2 to +8 °C for no longer than 48 h or at −80 °C for long-term storage. Repeated freeze–thaw cycles were not allowed.

After plasma separation, the erythrocyte pellet was washed three times with 0.9% isotonic sodium chloride solution at 4 °C using a 1:3 erythrocyte-to-saline ratio. After each wash, the suspension was centrifuged at 800× *g* for 5 min at 4 °C. To prepare the hemolysate, distilled water was added to the washed erythrocyte mass at a ratio of 1:9 (*v*/*v*) at 4 °C to induce hypotonic hemolysis. Hemoglobin (Hb) concentration was determined in parallel using the Drabkin method at 540 nm according to the manufacturer’s instructions. Purine parameters in the erythrocyte fraction were normalized to hemoglobin content and expressed as relative extinction units per gram of hemoglobin.

Pre-analytical quality control included exclusion of initial blood samples with marked hemolysis, lipemia, or icterus according to the laboratory visual scale; documentation of the time from blood collection to sample processing and freezing; and confirmation that repeated freeze–thaw cycles had not occurred. In summary, the principal pre-analytical timing constraints were plasma separation and aliquoting within 30 min of blood collection, serum separation after 30–45 min of clot formation, refrigerated storage at +2 to +8 °C for no longer than 48 h, and avoidance of repeated freeze–thaw cycles.

### 2.6. Hormonal Profile Assessment by ELISA

TSH was measured using the TSH AccuBind^®^ ELISA Test System (Monobind Inc., Lake Forest, CA, USA; Cat. No. 325-300A), based on a sandwich ELISA format. The manufacturer calibration range was 0–40 µIU/mL; samples exceeding the upper calibration limit were reassayed after appropriate dilution according to the manufacturer’s instructions. The manufacturer-reported analytical sensitivity was 0.078 µIU/mL after a 1 h incubation and 0.027 µIU/mL after a 2 h incubation. The reported intra-assay and inter-assay coefficients of variation (CVs) were 6.4–8.1% and 5.9–9.3%, respectively. Results were expressed in mIU/L, and the adult laboratory reference range was 0.4–4.0 mIU/L.

fT4 was measured using the Free T4 AccuBind^®^ ELISA Test System (Monobind Inc., USA; Cat. No. 1225-300A), based on a competitive ELISA format. The manufacturer’s calibration range extended to approximately 7.2 ng/dL, and the reported analytical sensitivity was 0.162 ng/dL. The manufacturer-reported intra-assay and inter-assay CVs were 3.25–10.98% and 6.01–10.81%, respectively. Results were expressed in pmol/L, and the laboratory reference range was 10–22 pmol/L.

Anti-TPO was measured using the Anti-TPO AccuBind^®^ ELISA Test System (Monobind Inc., USA; Cat. No. 1125-300A). The manufacturer’s calibration range was 0–500 IU/mL, with reassay after appropriate dilution for samples exceeding the upper calibration limit. The reported analytical sensitivity was 0.92 IU/mL, with intra-assay and inter-assay CVs of 3.8–5.7% and 4.5–6.8%, respectively.

Anti-TG was measured using the Anti-Tg AccuBind^®^ ELISA Test System (Monobind Inc., USA; Cat. No. 1025-300A). The manufacturer’s calibration range was 0–2000 IU/mL, with reassay after appropriate dilution for samples exceeding the upper calibration limit. The reported analytical sensitivity was 1.94 IU/mL, with intra-assay and inter-assay CVs of 3.6–5.0% and 4.0–5.3%, respectively. Anti-TPO and anti-TG results were expressed in IU/mL.

Absorbance for all ELISA assays was measured at 450 nm using a Thermo Scientific Multiskan FC microplate photometer (Thermo Fisher Scientific Oy, Vantaa, Finland; Cat. No. 51119000; spectral bandwidth 3–9 nm).

For pre-analytical processing, serum was obtained in tubes containing a clot activator and separating gel. After clot formation, samples were centrifuged and serum was separated. Analyses were performed using fresh material or aliquots stored at +2 to +8 °C for no longer than 48 h or at −80 °C without repeated freeze–thaw cycles.

Each analytical plate included two levels of internal control material, each analyzed in duplicate. The within-plate coefficient of variation for duplicate measurements was required to be ≤10%, and the inter-plate CV for control samples was required to be ≤15%. Calibration curves were accepted only when the coefficient of determination was R^2^ ≥ 0.995. An analytical run was accepted only when both levels of internal control material were within their predefined acceptance ranges and all applicable precision and calibration criteria were fulfilled. Plates failing any of these criteria were rejected and the analysis was repeated.

Free triiodothyronine (fT3) was not included in the original analytical protocol and was therefore not available for retrospective analysis. Thyroid functional phenotypes in the present study were classified using serum TSH and fT4. This approach is consistent with clinical recommendations for the biochemical evaluation of primary hypothyroidism, in which TSH and fT4 are the principal diagnostic parameters, whereas serum triiodothyronine measurement is not required for the diagnosis of hypothyroidism [30].

### 2.7. Determination of Purine Metabolites in Plasma and Erythrocytes

Purine metabolism parameters in plasma and the erythrocyte fraction were determined by direct ultraviolet spectrophotometry according to the method described by Oreshnikov et al. [31] and subsequently applied in studies of intermediate purine metabolites [32].

A 0.30 mL aliquot of plasma or prepared erythrocyte hemolysate was placed into a standard glass tube and subjected to thermal protein coagulation in a boiling water bath for 5 min. After cooling, 3.0 mL of bidistilled water was added, and the sample was incubated for 30 min at 37 °C.

Absorbance measurements were performed using a UV-1800 UV–Vis spectrophotometer (Shimadzu Corporation, Kyoto, Japan) with a spectral bandwidth of 1 nm, using quartz cuvettes with a 10 mm optical path length. The absorbance of the aqueous extracts was measured at 246 nm for guanine, 250 nm for hypoxanthine, 261 nm for adenine, 276 nm for xanthine, and 293 nm for uric acid, in accordance with the applied spectrophotometric methodology. At each wavelength, bidistilled water, corresponding to the pure extractant used in the analytical procedure, served as the reference blank. This background correction accounted for the absorbance of the extractant and the instrumental baseline but did not constitute matrix-specific spectral correction or separation of co-absorbing compounds. Accordingly, spectral overlap from endogenous UV-absorbing matrix constituents cannot be excluded, and matrix effects may contribute to the measured absorbance at the selected wavelengths.

For the comparative analyses reported in the present study, all purine parameters, including uric acid, were expressed on the same relative extinction scale. No conversion of the reported purine values to absolute molar concentrations was applied. Accordingly, plasma purine parameters were expressed in relative extinction units.

Preparation of the erythrocyte fraction and hemolysate was performed as described in Section 2.5. Hemoglobin concentration was determined in parallel using the cyanmethemoglobin/Drabkin method at 540 nm according to the manufacturer’s instructions. Erythrocyte purine parameters were normalized to hemoglobin content and expressed as relative extinction units per gram of hemoglobin. Hemoglobin normalization was used to standardize erythrocyte measurements between samples and did not represent conversion of the spectrophotometric values to absolute molar concentrations.

The X/HX, UA/X, and UA/HX ratios were calculated as literature-based relative indices of purine metabolism. The biological rationale for these ratios is based on the sequential conversion of HX to X and X to UA and on previous human pharmacodynamic studies demonstrating that purine product-to-substrate ratios respond to pharmacological inhibition of XO. Previous studies have used endogenous UA/X and X/HX ratios as indirect proxies of XO-related purine metabolism in human plasma. In the present study, however, X/HX, UA/X, and UA/HX were treated as exploratory indices of the relative distribution of sequential purine metabolites and were not interpreted as measures of XO/XOR enzymatic activity. These ratios do not constitute direct measurements of metabolic flux or XOR/XO/XDH enzymatic activity, cannot distinguish XDH from XO activity, and do not establish XOR-derived ROS generation. The indices were calculated as follows:

X/HX = X/HX

UA/X = UA/X

UA/HX = UA/HX

For each ratio, the numerator and denominator were obtained using the same sample-preparation and spectrophotometric framework and were reported in relative extinction-based units within the corresponding biological matrix. The calculated indices are dimensionless ratios of method-specific spectrophotometric signals. No wavelength-specific molar absorptivity coefficients (ε) or analyte-specific calibration factors were applied to convert the individual extinction signals into molar concentrations. Therefore, ratios derived from signals measured at different wavelengths, such as A276/A250, do not represent molar product-to-substrate ratios and should not be interpreted as stoichiometric concentration ratios or quantitative measures of metabolic flux.

No chromatographic separation or formal cross-validation against high-performance liquid chromatography with ultraviolet detection (HPLC-UV) or liquid chromatography–tandem mass spectrometry (LC–MS/MS) was performed as part of the original analytical protocol. The study was designed for comparative assessment of relative extinction-based purine-metabolism parameters rather than for analyte-specific absolute quantification. Because paired chromatographic measurements were not obtained from the same study specimens, a formal retrospective method-comparison analysis could not be performed. Accordingly, the present measurements should be interpreted within the analytical framework of the applied direct UV-spectrophotometric method and not as chromatographically resolved analyte-specific concentrations. Routine technical repeat measurements were used to monitor analytical consistency but cannot substitute for chromatographic validation of analyte selectivity.

### 2.8. Determination of MDA

Lipid peroxidation was assessed using the TBARS assay, with results expressed as MDA equivalents.

For plasma MDA determination, a plasma aliquot was used. For erythrocyte MDA determination, washed erythrocytes were subjected to hypotonic lysis, and the resulting hemolysate was analyzed. Buffer (0.15 M Tris-HCl, pH 7.4) was added to the sample aliquot to a final volume of 250 µL, followed by the addition of 500 µL of thiobarbituric acid (TBA) reagent (0.3% *w*/*v* in 15% *w*/*v* trichloroacetic acid [TCA]). Samples were mixed and boiled for 60 min in a water bath. No exogenous antioxidant or metal-chelating agent, including butylated hydroxytoluene, ethylenediaminetetraacetic acid, or deferoxamine, was added to the reaction mixture during sample preparation. Plasma and erythrocyte samples were processed using the same standardized analytical protocol.

After cooling, samples were centrifuged for 10 min at 6000 rpm, and the absorbance of the supernatant was measured at 540 nm using the UV-1800 UV–Vis spectrophotometer (Shimadzu Corporation, Kyoto, Japan; spectral bandwidth 1 nm) in quartz cuvettes with a 10 mm optical path length, against a blank control containing water/buffer with reagents but no sample. Quantitative assessment was performed using a calibration curve prepared with 1,1,3,3-tetramethoxypropane (TMP), and results were expressed as µmol/L for plasma and nmol/g Hb for erythrocytes [33,34].

Hemoglobin concentration in the hemolysate was determined by the cyanmethemoglobin method using Drabkin’s reagent. Samples were incubated with Drabkin’s reagent to form stable cyanmethemoglobin, and absorbance was measured at 540 nm using the same UV-1800 UV–Vis spectrophotometer described above. The obtained hemoglobin values were used to normalize erythrocyte TBARS-derived MDA results, which were expressed as nmol/g Hb [35].

Calibration curves prepared using TMP were accepted only when the coefficient of determination was R^2^ ≥ 0.995. Plasma and erythrocyte samples were analyzed in duplicate, with a within-run coefficient of variation between duplicate measurements required to be ≤10%, and two levels of internal control material were included in each analytical series. The same analytical quality-control criteria were applied to both biological matrices. Formal matrix-specific intra-assay and inter-assay CVs were not separately established as validation parameters in the original study and therefore are not retrospectively reported. Initial samples showing marked pre-analytical hemolysis or lipemia were excluded.

### 2.9. Statistical Analysis

Statistical analysis was performed using GraphPad Prism version 8.0.1 (GraphPad Software, San Diego, CA, USA). Quantitative variables were summarized using the median and interquartile range, median [Q1–Q3]. When appropriate, mean values and standard deviations were additionally reported as mean ± SD. The predefined group sizes were maintained throughout the laboratory and statistical analyses: hypothyroidism without AIT (*n* = 31), AIT with hypothyroidism (*n* = 30), nontoxic nodular goiter with euthyroidism (*n* = 42), euthyroid AIT (*n* = 37), and healthy controls (*n* = 24).

Between-group differences among the five independent groups were assessed using the Kruskal–Wallis test. When the overall Kruskal–Wallis test was statistically significant, post hoc pairwise comparisons were performed using Dunn’s multiple-comparisons test with correction for multiple comparisons, as implemented in GraphPad Prism. Statistical significance was set at *p* < 0.05, and numerical *p* values were reported whenever available.

For X/HX, UA/X, and UA/HX, each ratio was calculated separately for each participant from the corresponding individual purine measurements within the same biological matrix before group-level summarization. Participant-level ratios were summarized as median [Q1–Q3] and compared among the five groups using the Kruskal–Wallis test. When the overall Kruskal–Wallis test was not statistically significant, post hoc comparisons were not interpreted as evidence of between-group differences. Percentage differences reported in the Results were calculated from group medians for descriptive purposes; statistical significance of the corresponding pairwise comparisons was determined using Dunn’s test. Between-group analyses of the participant-level X/HX, UA/X, and UA/HX ratios were treated as the principal inferential analyses, whereas the correlation analyses were considered exploratory.

Associations between participant-level purine-metabolite ratios and MDA levels were assessed using standard unadjusted bivariate Spearman rank correlation analysis. Plasma and erythrocyte X/HX, UA/HX, and UA/X ratios were analyzed separately in relation to plasma and erythrocyte MDA. Results are reported as Spearman’s rank correlation coefficient (ρ), 95% confidence interval, and two-sided *p*-value. All correlation analyses were performed on the full analytic sample (*n* = 164), with no missing observations for the variables included in these analyses. No partial Spearman correlation, rank-based regression, or multivariable covariate-adjusted regression was performed. Because the study was designed as an exploratory cross-sectional analysis, multivariable modeling was not prespecified in the original statistical plan. In addition, inflammatory markers were not systematically available across the cohort, and levothyroxine exposure was strongly linked to the hypothyroid clinical phenotypes. Therefore, the correlation analyses were retained as unadjusted exploratory analyses, and no claims of independent covariate-adjusted associations were made. Correlation analyses were considered exploratory, with *p* < 0.05 regarded as statistically significant.

Levothyroxine exposure, weight-adjusted dose, and treatment duration were summarized descriptively. Treatment-stratified analyses were not prespecified and were not performed because the untreated subgroups within the two hypothyroid phenotypes were small and treatment assignment was clinically determined rather than randomized, limiting the interpretability of post hoc treated-versus-untreated comparisons.

## 3. Results

### 3.1. Baseline Demographic, Clinical, Hormonal, and Renal Characteristics

The study included 164 participants distributed among five predefined clinical groups: hypothyroidism without AIT (*n* = 31), AIT with hypothyroidism (*n* = 30), nontoxic nodular goiter with euthyroidism (*n* = 42), euthyroid AIT (*n* = 37), and healthy controls (*n* = 24). Detailed baseline demographic, anthropometric, hormonal, immunological, and renal characteristics of the study groups are presented in Table 1.

Mean age was 41.19 ± 7.97 years in the hypothyroidism-without-AIT group, 41.03 ± 8.95 years in the AIT-with-hypothyroidism group, 39.93 ± 8.50 years in the nontoxic nodular goiter group, 43.65 ± 8.90 years in the euthyroid-AIT group, and 40.54 ± 8.66 years in the control group. The corresponding median ages were 41 (34–47), 42.5 (33.3–48.0), 39 (33–46), 44 (38–51), and 40 (35.8–44.5) years, respectively. Women predominated in all five groups and accounted for 80.6%, 86.7%, 76.2%, 83.8%, and 66.7% of participants, respectively.

Median body mass index (BMI) was 29.3 (27.1–31.8) kg/m^2^ in participants with hypothyroidism without AIT, 26.2 (24.7–29.1) kg/m^2^ in the AIT-with-hypothyroidism group, 27.9 (25.3–29.3) kg/m^2^ in participants with nontoxic nodular goiter, 26.2 (24.8–29.0) kg/m^2^ in euthyroid AIT, and 25.8 (23.5–27.0) kg/m^2^ in healthy controls.

The thyroid hormonal profiles reflected the predefined functional classification of the study groups. Median TSH concentrations were 7.95 (5.16–11.66) mIU/L in hypothyroidism without AIT and 8.52 (5.89–13.79) mIU/L in AIT with hypothyroidism. In the euthyroid groups, median TSH concentrations were 2.19 (1.71–2.66) mIU/L in nontoxic nodular goiter, 2.00 (1.35–2.61) mIU/L in euthyroid AIT, and 1.85 (1.45–2.08) mIU/L in healthy controls. Median fT4 concentrations were 12.3 (8.6–13.7), 11.8 (8.5–13.6), 16.3 (14.4–17.2), 14.2 (13.2–15.9), and 15.7 (15.1–17.0) pmol/L in the five groups, respectively.

As expected from the clinical phenotype definitions, thyroid autoantibody titers were substantially higher in the two AIT groups. Median anti-TPO concentrations were 221.0 (162.7–415.3) IU/mL in AIT with hypothyroidism and 170.2 (81.0–369.4) IU/mL in euthyroid AIT, compared with 14.5 (8.7–20.0) IU/mL in hypothyroidism without AIT, 15.3 (9.8–18.7) IU/mL in nontoxic nodular goiter, and 11.5 (8.3–16.2) IU/mL in controls. Median anti-TG concentrations were 213.9 (112.9–269.0) IU/mL and 218.6 (119.5–408.4) IU/mL in the two AIT groups, respectively, compared with 41.3 (31.1–67.0), 36.1 (22.9–57.5), and 36.2 (24.0–45.2) IU/mL in hypothyroidism without AIT, nontoxic nodular goiter, and controls, respectively.

Within the hypothyroidism-without-AIT group, 20 of 31 participants (64.5%) had subclinical hypothyroidism and 11 (35.5%) had overt hypothyroidism. In the AIT-with-hypothyroidism group, 18 of 30 participants (60.0%) had subclinical hypothyroidism and 12 (40.0%) had overt hypothyroidism. Thus, both hypothyroid groups contained participants with subclinical and overt disease, with broadly similar proportions of the two functional categories.

Levothyroxine replacement therapy was used by 22 of 31 participants (71.0%) in the hypothyroidism-without-AIT group and by 24 of 30 participants (80.0%) in the AIT-with-hypothyroidism group. Among participants receiving L-T4, the median weight-adjusted daily dose was 1.00 (0.73–1.28) µg/kg/day in Group 1 and 0.91 (0.73–1.18) µg/kg/day in Group 2, while the corresponding median treatment durations were 39.5 (23.8–70.5) and 46.5 (26.0–74.3) months, respectively. Participants in the euthyroid groups and healthy controls were not receiving levothyroxine.

Renal function was additionally characterized because renal clearance is an important determinant of circulating uric acid and may influence the distribution of other purine metabolites. Median estimated glomerular filtration rate (eGFR) was 91.4 (84.2–104.6) mL/min/1.73 m^2^ in hypothyroidism without AIT, 92.5 (82.8–99.1) mL/min/1.73 m^2^ in AIT with hypothyroidism, 101.1 (88.6–112.5) mL/min/1.73 m^2^ in nontoxic nodular goiter, 96.9 (84.0–104.0) mL/min/1.73 m^2^ in euthyroid AIT, and 100.5 (95.6–114.2) mL/min/1.73 m^2^ in healthy controls. Median eGFR was above 90 mL/min/1.73 m^2^ in all five groups, indicating generally preserved glomerular filtration at the group level. Nevertheless, because eGFR does not directly characterize renal tubular handling of urate and other purine metabolites, renal contribution to interindividual variability cannot be completely excluded. Age, sex, BMI, eGFR, and L-T4 exposure, including weight-adjusted dose and treatment duration, are reported in Table 1 because these variables represent clinically relevant potential sources of variability in purine-metabolism and oxidative-stress parameters.

### 3.2. Plasma Purine-Metabolite Parameters

Plasma purine-metabolite profiles differed across the five study groups, with the clearest phenotype-related differences observed for guanine and hypoxanthine, whereas adenine, xanthine, and uric acid showed more selective between-group patterns (Table 2).

Because these metabolites were assessed by direct UV spectrophotometry and reported in relative extinction units, the values described below represent relative spectrophotometric signals rather than absolute concentrations of the individual purine metabolites.

Plasma guanine showed the most pronounced overall between-group difference (H(4) = 29.66, *p* < 0.0001). The median guanine level in the AIT-with-hypothyroidism group was 40.3% lower than in patients with hypothyroidism without AIT (*p* = 0.0115), 35.4% lower than in patients with nontoxic nodular goiter (*p* < 0.0001), and 19.9% lower than in controls (*p* = 0.0035). In euthyroid AIT, the median guanine level was 32.5% lower than in nontoxic nodular goiter (*p* = 0.0019) and 16.3% lower than in controls (*p* = 0.0427). The two AIT groups did not differ significantly from each other (*p* > 0.9999). Thus, lower plasma guanine values were observed in both AIT groups relative to selected clinical comparator groups; however, these comparisons do not establish an independent effect of autoimmune status because the euthyroid comparator group consisted of patients with nontoxic nodular goiter rather than otherwise healthy euthyroid autoantibody-negative participants.

Plasma hypoxanthine also differed significantly among the five groups (H(4) = 28.78, *p* < 0.0001). In the AIT-with-hypothyroidism group, the median hypoxanthine level was 37.6% lower than in patients with hypothyroidism without AIT (*p* = 0.0316), 39.9% lower than in patients with nontoxic nodular goiter (*p* < 0.0001), and 22.3% lower than in controls (*p* = 0.0067). In euthyroid AIT, the median hypoxanthine level was 38.3% lower than in nontoxic nodular goiter (*p* = 0.0015). Although the median value in euthyroid AIT was 20.3% lower than in controls, this comparison did not reach statistical significance (*p* = 0.0904). No significant difference was observed between the two AIT groups (*p* > 0.9999). These findings demonstrate phenotype-related differences in plasma hypoxanthine; however, they should not be interpreted as evidence of an independent autoimmune effect across both thyroid functional strata.

Plasma adenine also differed significantly among the five groups (H(4) = 23.04, *p* = 0.0001), but its pattern was more phenotype-specific. The highest median adenine level was observed in Nontoxic nodular goiter. Compared with this group, adenine was 40.1% lower in AIT with hypothyroidism (*p* = 0.0003) and 36.4% lower in euthyroid AIT (*p* = 0.0026). In contrast, the direct comparison between the AIT-with-hypothyroidism and hypothyroidism-without-AIT groups was not significant (*p* = 0.4314), and neither AIT group differed significantly from controls (*p* = 0.0694 and *p* = 0.3665, respectively). Therefore, unlike guanine and hypoxanthine, the adenine findings do not support a uniform AIT-associated difference across both thyroid functional states.

Significant overall heterogeneity was also found for plasma xanthine (H(4) = 16.84, *p* = 0.0021). The highest median xanthine level occurred in Nontoxic nodular goiter. Relative to this group, xanthine was 21.9% lower in AIT with hypothyroidism (*p* = 0.0022) and 17.5% lower in euthyroid AIT (*p* = 0.0361). No statistically significant differences were observed between any of the clinical groups and controls. In particular, the direct comparison between the AIT-with-hypothyroidism and hypothyroidism-without-AIT groups was not significant (*p* > 0.9999). Thus, the xanthine pattern was driven primarily by differences involving the Nontoxic nodular goiter group rather than by a uniform shift relative to healthy controls.

Plasma uric acid likewise differed significantly across the five groups (H(4) = 13.56, *p* = 0.0088). Post hoc analysis identified only one significant pairwise comparison: the median uric acid level in AIT with hypothyroidism was 12.8% lower than in Nontoxic nodular goiter (*p* = 0.0174). No other pairwise comparisons were statistically significant, including comparisons between the clinical groups and controls. Accordingly, although overall heterogeneity was present, the data do not indicate a consistent increase or decrease in plasma uric acid associated with AIT or thyroid functional status alone.

Overall, significant between-group heterogeneity was observed for all five plasma purine metabolites. The clearest differences involved guanine and hypoxanthine: both were lower in the AIT-with-hypothyroidism group than in the hypothyroidism-without-AIT group and were also lower in euthyroid AIT than in nontoxic nodular goiter. Adenine, xanthine, and uric acid showed more selective phenotype-dependent differences, many of which involved the nontoxic nodular goiter group. The findings therefore demonstrate distinct plasma purine-metabolite profiles across the predefined clinical phenotypes but do not establish a uniform AIT-specific metabolic pattern (Figure 1).

### 3.3. Erythrocyte Levels of Purine Metabolites

Erythrocyte purine-metabolite profiles showed a broad elevation across thyroid-disease groups relative to healthy controls, with comparatively limited separation among the four clinical thyroid phenotypes (Table 3). As in plasma, erythrocyte purine-metabolite values represent relative extinction-based spectrophotometric signals normalized to hemoglobin and should not be interpreted as absolute intracellular metabolite concentrations.

Erythrocyte guanine differed significantly among the five groups (H(4) = 30.59, *p* < 0.0001). Compared with the control group, the median guanine level was 29.7% higher in hypothyroidism without AIT (*p* = 0.0006), 15.4% higher in AIT with hypothyroidism (*p* = 0.0152), 56.3% higher in Nontoxic nodular goiter (*p* < 0.0001), and 19.8% higher in euthyroid AIT (*p* = 0.0005). Despite these consistent differences from controls, no statistically significant pairwise differences were observed among the four clinical groups. Thus, the erythrocyte guanine pattern was characterized by a generalized elevation in thyroid pathology rather than by an AIT-specific or thyroid-function-specific difference.

A similar overall pattern was observed for erythrocyte hypoxanthine (H(4) = 26.13, *p* < 0.0001). Relative to controls, the median hypoxanthine level was 30.5% higher in hypothyroidism without AIT (*p* = 0.0030), 17.7% higher in AIT with hypothyroidism (*p* = 0.0698), 57.1% higher in Nontoxic nodular goiter (*p* < 0.0001), and 26.9% higher in euthyroid AIT (*p* = 0.0033). The increase in AIT with hypothyroidism did not reach statistical significance, whereas the other three clinical groups differed significantly from controls. No significant differences were detected among the clinical groups themselves.

Erythrocyte adenine also showed marked overall between-group heterogeneity (H(4) = 28.84, *p* < 0.0001). The median adenine level was 35.4% higher in hypothyroidism without AIT than in controls (*p* = 0.0046), 25.9% higher in AIT with hypothyroidism (*p* = 0.0428), 59.1% higher in Nontoxic nodular goiter (*p* < 0.0001), and 35.4% higher in euthyroid AIT (*p* = 0.0014). As for guanine, all four clinical groups therefore showed significantly higher erythrocyte adenine than controls, whereas no statistically significant differences were observed among the four thyroid-disease phenotypes.

The most pronounced overall between-group heterogeneity among the erythrocyte purine metabolites was observed for xanthine (H(4) = 32.29, *p* < 0.0001). Compared with controls, the median xanthine level was 45.2% higher in hypothyroidism without AIT (*p* < 0.0001), 35.2% higher in AIT with hypothyroidism (*p* = 0.0384), 66.1% higher in Nontoxic nodular goiter (*p* < 0.0001), and 42.6% higher in euthyroid AIT (*p* = 0.0009). All four clinical groups differed significantly from controls, while none differed significantly from another clinical group. Therefore, erythrocyte xanthine showed a broad disease-associated elevation rather than a phenotype-specific pattern.

Erythrocyte uric acid likewise differed significantly among the five groups (H(4) = 32.55, *p* < 0.0001). Compared with controls, the median uric acid level was 55.0% higher in hypothyroidism without AIT (*p* = 0.0002), 21.9% higher in AIT with hypothyroidism (*p* = 0.1736), 61.3% higher in Nontoxic nodular goiter (*p* < 0.0001), and 28.7% higher in euthyroid AIT (*p* = 0.0027). The difference between AIT with hypothyroidism and controls was not statistically significant, whereas the other three clinical groups showed significant elevations. No significant differences in erythrocyte uric acid were identified among the four clinical phenotypes.

Overall, the erythrocyte data demonstrated a pattern that differed substantially from the plasma profile. All five erythrocyte purine-metabolite parameters showed significant overall between-group heterogeneity. Guanine, adenine, and xanthine were significantly higher in all four thyroid-disease groups than in controls, whereas hypoxanthine and uric acid were significantly higher in three of the four clinical groups, with the same numerical direction also observed in AIT with hypothyroidism. Importantly, none of the five erythrocyte purine-metabolite parameters showed significant pairwise differences among the four clinical groups. The erythrocyte findings therefore indicate a broad elevation of relative purine-metabolite values across thyroid-disease groups compared with controls, without consistent separation among the four clinical phenotypes (Figure 2).

### 3.4. Relative Purine-Metabolite Indices

The X/HX, UA/X, and UA/HX ratios were calculated at the individual-participant level before group-level summarization. Analysis of these ratios revealed distinct patterns in plasma and erythrocytes, with the most pronounced between-group differences involving plasma X/HX and UA/HX and erythrocyte X/HX.

In plasma, X/HX differed significantly among the five groups (H(4) = 22.84, *p* = 0.0001). Relative to the control group, the median X/HX ratio was 14.7% higher in the AIT-with-hypothyroidism group (*p* = 0.0005) and 14.4% higher in the euthyroid AIT group (*p* = 0.0005). By contrast, the corresponding increases relative to the control group were 13.5% in hypothyroidism without AIT (*p* = 0.4600) and 4.3% in Nontoxic nodular goiter (*p* = 0.2178); neither difference was statistically significant. Direct comparison of AIT and non-AIT phenotypes within the same thyroid functional state showed that X/HX was only 1.0% higher in AIT with hypothyroidism than in hypothyroidism without AIT (*p* = 0.4089) and 9.7% higher in euthyroid AIT than in Nontoxic nodular goiter (*p* = 0.4833). Thus, the principal statistical feature of plasma X/HX was the separation of both AIT groups from healthy controls rather than a significant separation of AIT from non-AIT participants with the same thyroid functional status (Table 4).

A similar but quantitatively more pronounced pattern was observed for plasma UA/HX (H(4) = 19.08, *p* = 0.0008). Compared with the control group, the median UA/HX ratio was 21.5% higher in AIT with hypothyroidism (*p* = 0.0038) and 19.9% higher in euthyroid AIT (*p* = 0.0022). In comparison, hypothyroidism without AIT showed only a 3.3% increase relative to the control group (*p* = 0.5218), whereas Nontoxic nodular goiter showed a 10.7% increase (*p* > 0.9999); neither difference was statistically significant. Within the same thyroid functional categories, UA/HX was numerically 17.7% higher in AIT with hypothyroidism than in hypothyroidism without AIT (*p* > 0.9999) and 8.3% higher in euthyroid AIT than in Nontoxic nodular goiter (*p* = 0.2211). Accordingly, the statistically supported plasma UA/HX pattern, as for X/HX, was defined primarily by higher values in the AIT groups relative to healthy controls, whereas direct comparisons between corresponding AIT and non-AIT phenotypes were not significant.

Plasma UA/X showed substantially less between-group separation. Compared with the control group, the median UA/X ratio was 7.7% higher in hypothyroidism without AIT (*p* = 0.1072), 8.9% higher in AIT with hypothyroidism (*p* = 0.6875), 6.8% higher in Nontoxic nodular goiter (*p* > 0.9999), and 9.2% higher in euthyroid AIT (*p* = 0.2551). None of these differences was statistically significant. Within the corresponding thyroid functional categories, the presence of AIT was associated with only a 1.1% higher median UA/X in hypothyroidism (*p* > 0.9999) and a 2.2% higher median UA/X in euthyroidism (*p* > 0.9999). Consistent with these pairwise findings, the overall Kruskal–Wallis test did not demonstrate statistically significant between-group heterogeneity (H(4) = 9.064, *p* = 0.0595). Thus, unlike X/HX and UA/HX, plasma UA/X showed no statistically confirmed group differences (Figure 3).

The erythrocyte findings differed substantially from the plasma pattern. Significant heterogeneity was observed for erythrocyte X/HX (H(4) = 19.95, *p* = 0.0005), but this difference was driven by the hypothyroidism without AIT group rather than by the AIT groups. In the hypothyroidism-without-AIT group, the median X/HX ratio was 9.9% higher than in the control group (*p* = 0.0331), 13.5% higher than in the AIT-with-hypothyroidism group (*p* = 0.0009), 10.0% higher than in nontoxic nodular goiter (*p* = 0.0119), and 10.3% higher than in euthyroid AIT (*p* = 0.0016). In contrast, the other thyroid-disease groups remained close to the control value: erythrocyte X/HX was 3.2% lower in AIT with hypothyroidism than in controls (*p* > 0.9999), 0.2% lower in Nontoxic nodular goiter (*p* > 0.9999), and 0.4% lower in euthyroid AIT (*p* > 0.9999). These findings identify hypothyroidism without AIT as the phenotype responsible for the significant erythrocyte X/HX heterogeneity.

Neither of the remaining erythrocyte ratios showed statistically significant between-group differences. For UA/X, compared with the control group, the median value was 3.6% lower in hypothyroidism without AIT (*p* > 0.9999), 4.9% lower in AIT with hypothyroidism (*p* > 0.9999), 7.8% lower in Nontoxic nodular goiter (*p* > 0.9999), and 4.2% lower in euthyroid AIT (*p* > 0.9999). The distributions overlapped extensively, and the overall Kruskal–Wallis test was clearly non-significant (H(4) = 1.119, *p* = 0.8913). Thus, the modest numerical variation in erythrocyte UA/X did not represent a statistically supported group effect.

Similarly, erythrocyte UA/HX showed numerical variation without statistically significant between-group heterogeneity (H(4) = 7.853, *p* = 0.0971). Relative to the control group, the median UA/HX ratio was 11.7% higher in hypothyroidism without AIT (*p* > 0.9999), whereas it was 8.4% lower in AIT with hypothyroidism (*p* > 0.9999), 8.4% lower in Nontoxic nodular goiter (*p* > 0.9999), and 8.1% lower in euthyroid AIT (*p* > 0.9999). These numerical differences should therefore not be interpreted as statistically established group effects (Figure 4).

Taken together, the participant-level analysis demonstrates a clear compartment-specific pattern. In plasma, the statistically supported differences were confined to X/HX and UA/HX and were characterized by higher values in both AIT groups relative to healthy controls. However, the absence of significant AIT-versus-non-AIT differences within the same thyroid functional categories indicates that these findings should not be interpreted as evidence of an independent effect of autoimmune status. In erythrocytes, the pattern was different: only X/HX differed significantly, and the difference was driven specifically by higher values in hypothyroidism without AIT. UA/X showed no significant group differences in either biological compartment, and erythrocyte UA/HX was likewise non-significant. Overall, these findings indicate phenotype- and compartment-dependent differences in the relative distribution of sequential purine metabolites, without providing direct evidence of altered reaction rates, metabolic flux, or XOR/XO/XDH enzymatic activity.

### 3.5. TBARS-Derived MDA Equivalents as an Index of Lipid Peroxidation

Lipid peroxidation was assessed using TBARS-derived MDA equivalents in plasma and erythrocytes. Plasma values were expressed in µmol/L, whereas erythrocyte values were normalized to hemoglobin and expressed in nmol/g Hb. Data are presented as median [Q1–Q3]. Overall between-group differences were assessed using the Kruskal–Wallis test, followed by Dunn’s multiple-comparisons test for pairwise comparisons (Table 5).

Plasma MDA differed markedly among the five study groups (H(4) = 59.87, *p* < 0.0001). The control group had the lowest median plasma MDA level, at 1.244 μmol/L. Compared with controls, plasma MDA was 23.4% higher in hypothyroidism without AIT (1.535 μmol/L; *p* < 0.0001), 22.3% higher in AIT with hypothyroidism (1.522 μmol/L; *p* < 0.0001), 29.8% higher in Nontoxic nodular goiter (1.615 μmol/L; *p* < 0.0001), and 23.4% higher in euthyroid AIT (1.535 μmol/L; *p* < 0.0001). In contrast, none of the pairwise comparisons among the four clinical groups was statistically significant (all *p* > 0.9999). Thus, the plasma MDA profile was characterized by a generalized elevation across thyroid-pathology phenotypes rather than by a difference specifically associated with AIT status or thyroid functional state.

Erythrocyte MDA also differed significantly among the five groups (H(4) = 33.82, *p* < 0.0001), but the pattern was distinctly different from that observed in plasma. The highest median erythrocyte MDA level was observed in hypothyroidism without AIT, at 13.97 nmol/g Hb, which was 66.4% higher than the control value of 8.397 nmol/g Hb (*p* < 0.0001). Compared with hypothyroidism without AIT, erythrocyte MDA was 34.4% lower in AIT with hypothyroidism (*p* = 0.0059), 29.8% lower in Nontoxic nodular goiter (*p* = 0.0039), and 33.9% lower in euthyroid AIT (*p* = 0.0046).

Relative to controls, median erythrocyte MDA was 9.2% higher in AIT with hypothyroidism (*p* = 0.2404), 16.8% higher in Nontoxic nodular goiter (*p* = 0.0888), and 9.9% higher in euthyroid AIT (*p* = 0.1301); however, none of these differences was statistically significant. Furthermore, AIT with hypothyroidism, Nontoxic nodular goiter, and euthyroid AIT did not differ significantly from one another (all *p* > 0.9999).

Overall, the MDA results demonstrate a clear compartment-specific pattern of lipid peroxidation. Plasma MDA was consistently and significantly elevated in all four thyroid-disease groups relative to controls, without significant differences among the clinical phenotypes. In contrast, erythrocyte MDA showed a selective and pronounced elevation in hypothyroidism without AIT, whereas the remaining three clinical groups did not differ significantly from controls. Thus, circulating and erythrocyte lipid-peroxidation profiles were not parallel across the investigated thyroid phenotypes (Figure 5).

### 3.6. Correlation Analysis

Spearman rank correlation analysis was performed to assess associations between participant-level purine-metabolite ratios and TBARS-derived MDA equivalents in plasma and erythrocytes. In the primary pooled analysis, plasma and erythrocyte X/HX, UA/HX, and UA/X ratios were analyzed separately in relation to plasma and erythrocyte MDA in the full analytic cohort (Table 6).

The strongest pooled association was observed between plasma UA/X and plasma MDA (ρ = 0.376, 95% CI 0.237–0.501, *p* = 6.86 × 10^−7^). Plasma UA/X was also positively associated with erythrocyte MDA (ρ = 0.210, 95% CI 0.059–0.352, *p* = 0.0068).

Plasma UA/HX showed a weaker positive association with plasma MDA (ρ = 0.228, 95% CI 0.078–0.369, *p* = 0.0033), whereas its association with erythrocyte MDA was not statistically significant (ρ = 0.134, 95% CI −0.020–0.281, *p* = 0.0873). Plasma X/HX was not significantly associated with either plasma or erythrocyte MDA.

Among the erythrocyte ratios, erythrocyte X/HX showed a weak inverse association with plasma MDA (ρ = −0.162, 95% CI −0.307 to −0.009, *p* = 0.0387), whereas no significant association was observed with erythrocyte MDA. Erythrocyte UA/HX and UA/X were not significantly associated with either plasma or erythrocyte MDA.

To examine whether the pooled associations involving the uric-acid-containing ratios could be driven by separation of the diagnostic clusters, additional Spearman correlation analyses were performed separately within each of the five predefined clinical groups. These analyses evaluated plasma UA/HX and UA/X in relation to plasma and erythrocyte MDA (Table 7).

The group-stratified analysis demonstrated that the pooled associations were not uniform across thyroid phenotypes. The positive association between plasma UA/X and plasma MDA was reproduced within hypothyroidism without AIT (ρ = 0.418, *p* = 0.0194), AIT with hypothyroidism (ρ = 0.867, *p* < 0.0001), and euthyroid AIT (ρ = 0.687, *p* < 0.0001), whereas no corresponding association was observed in nontoxic nodular goiter with euthyroidism or healthy controls.

A different pattern was observed for plasma UA/HX. Its association with plasma MDA was inverse in hypothyroidism without AIT (ρ = −0.599, *p* = 0.0004), but positive in AIT with hypothyroidism (ρ = 0.552, *p* = 0.0016) and euthyroid AIT (ρ = 0.494, *p* = 0.0019). No association was observed in nontoxic nodular goiter with euthyroidism, while the inverse association in healthy controls did not reach statistical significance (ρ = −0.390, *p* = 0.0593).

Associations with erythrocyte MDA were substantially less consistent across phenotypes. Plasma UA/HX was not significantly associated with erythrocyte MDA within any individual group. For plasma UA/X, nominal positive associations with erythrocyte MDA were observed in hypothyroidism without AIT (ρ = 0.375, *p* = 0.0374) and in healthy controls (ρ = 0.405, *p* = 0.0498), whereas no corresponding associations were detected in the other three groups. Given the exploratory nature of the subgroup analyses and the absence of multiplicity adjustment, these latter findings should be interpreted cautiously.

Overall, the stratified results indicate that the pooled plasma UA/X–plasma MDA association cannot be explained solely by between-group separation, because a positive relationship was also observed within several thyroid-disease phenotypes. In contrast, the plasma UA/HX–plasma MDA relationship showed substantial phenotype-dependent variation in both magnitude and direction. Because these analyses were based on unadjusted bivariate correlations, the observed associations may also be influenced by age, sex, BMI, renal function, L-T4 exposure, and other clinical or metabolic factors. Accordingly, the correlation findings should be interpreted as exploratory associations rather than as independent covariate-adjusted relationships.

## 4. Discussion

The present study identified distinct plasma and erythrocyte patterns of purine-related parameters and lipid-peroxidation markers across the studied thyroid phenotypes. The analysis identified partially overlapping but non-equivalent plasma and erythrocyte profiles rather than a single biochemical pattern determined exclusively by autoimmune status or thyroid functional impairment. Evaluation of participant-level purine ratios and between-group comparisons does not support a simple model in which AIT uniformly redirects purine metabolism toward terminal catabolism. Instead, individual metabolites, calculated ratios, and MDA showed distinct patterns according to phenotype and biological compartment. Purine-metabolism research likewise indicates that the diagnostic significance of individual metabolites depends on the analytical method, biological matrix, metabolic context, and pathways of production and elimination [36]. This interpretation is consistent with clinical evidence indicating that systemic abnormalities may overlap across autoimmune and non-autoimmune forms of thyroid dysfunction. Hemogram-derived inflammatory indices have been reported to be elevated across euthyroid Hashimoto thyroiditis, hypothyroid Hashimoto thyroiditis, and non-immunogenic hypothyroidism, whereas oxidative-stress abnormalities may remain detectable after biochemical correction of hypothyroidism [37,38]. Thus, low-grade immune-inflammatory activity may represent an additional biological factor contributing to redox and purine-metabolic variability across thyroid phenotypes.

The biochemical heterogeneity observed across the thyroid phenotypes may be interpreted in the context of their distinct pathophysiological backgrounds. Thyroid hormones are major regulators of mitochondrial biogenesis, oxidative phosphorylation, ATP production, and mitochondrial redox homeostasis; consequently, reduced thyroid-hormone signaling in hypothyroidism may alter cellular bioenergetics and nucleotide turnover, thereby contributing to changes in purine-metabolite distribution as well as susceptibility to oxidative imbalance [39].

In AIT, these metabolic effects occur in parallel with loss of immune tolerance, activation of cellular and humoral immune responses, chronic lymphocytic inflammation, and oxidative injury to thyrocytes, creating a biological setting in which immune activation and redox disturbance may jointly influence systemic metabolic profiles [40].

The nontoxic nodular goiter with euthyroid phenotype represents a different pathophysiological context: this disorder is generally characterized by preserved thyroid function and has a multifactorial pathogenesis involving genetic, environmental, and follicular-growth mechanisms rather than a single disturbance of thyroid-hormone production [41].

Importantly, oxidative remodeling is not restricted to autoimmune or functionally hypothyroid thyroid disease: increased ROS generation has also been demonstrated in benign thyroid lesions compared with normal thyroid tissue [42].

Accordingly, the distinct plasma purine pattern and increased plasma TBARS-derived MDA observed in the euthyroid, antibody-negative nontoxic nodular goiter group in the present study further argue against attributing the biochemical changes exclusively to thyroid-hormone deficiency or autoimmunity. Taken together, the findings are more consistent with phenotype-specific combinations of mitochondrial, immune-inflammatory, structural, metabolic, and redox mechanisms than with a single pathway common to all thyroid disorders.

The plasma purine profile showed the clearest phenotype-related differences for guanine and hypoxanthine, whereas adenine, xanthine, and uric acid displayed a more selective distribution across the clinical phenotypes. Importantly, these between-group differences refer to relative extinction-based spectrophotometric signals and therefore indicate comparative differences within the applied analytical framework rather than absolute changes in metabolite concentrations. Most clinical research linking purine metabolism to thyroid physiology has focused on uric acid rather than on the complete spectrum of circulating purine metabolites. Population-based studies have demonstrated associations between serum uric acid or hyperuricemia and thyroid hormone sensitivity or thyroid-stimulating hormone, while also showing that these relationships vary according to demographic, anthropometric, renal, and metabolic characteristics [43,44]. Such associations therefore cannot be generalized to the entire purine pathway. The present findings broaden this perspective by indicating that intermediate purine metabolites may carry information that is not reflected by uric acid alone.

Renal handling represents an important additional determinant of the circulating purine profile. Uric acid is freely filtered at the glomerulus and undergoes extensive proximal-tubular reabsorption and secretion; URAT1 (SLC22A12) and GLUT9 (SLC2A9) are major components of the renal urate reabsorptive pathway [45]. Thyroid hormones influence renal hemodynamics, glomerular filtration, tubular transport, and sodium–water and electrolyte homeostasis; consequently, thyroid dysfunction may modify urate retention and excretion independently of changes in purine production [46]. Parathyroid hormone provides an additional endocrine–renal link through its effects on calcium, phosphate, and sodium handling, and altered parathyroid function has been associated with disturbances of urate homeostasis [47]. These thyroid–parathyroid–renal interactions therefore provide a plausible physiological context for variation in circulating uric acid across thyroid phenotypes, although they were not directly assessed in the present study. In our cohort, median eGFR remained above 90 mL/min/1.73 m^2^ in all five groups, with median values ranging from 91.4 to 101.1 mL/min/1.73 m^2^. Thus, marked impairment of glomerular filtration is unlikely to provide a sufficient explanation for the observed between-group plasma purine patterns. Nevertheless, eGFR does not directly characterize tubular urate transport or transporter activity, and a contribution of altered tubular handling to interindividual variability cannot be excluded.

The comparatively lower plasma guanine and hypoxanthine values observed in the AIT phenotypes are noteworthy, particularly because similar differences were evident across different thyroid functional states. Nevertheless, these findings should not be interpreted as direct evidence of accelerated purine degradation. The relative abundance of an individual circulating purine metabolite may be influenced by synthesis, degradation, salvage, cellular release and uptake, transport, and elimination. Xanthine oxidoreductase biology further indicates that static purine-metabolite values cannot be equated directly with a particular XDH/XO activity state, whereas stable-isotope tracing is specifically used to resolve dynamic pathway utilization [48,49]. Accordingly, static hypoxanthine, xanthine, or uric-acid concentrations–and ratios derived from them–cannot by themselves define pathway flux or XOR proteoform activity.

The presence of plasma purine alterations in euthyroid AIT is also biologically relevant because it indicates that metabolic differences may be detectable in autoimmune thyroid disease even in the absence of overt thyroid hormone deficiency. Recent clinical evidence demonstrates altered oxidative balance in euthyroid women with Hashimoto thyroiditis, while a contemporary review identifies oxidative stress as an important component of Hashimoto thyroiditis pathophysiology [50,51]. Therefore, the plasma pattern observed in the present study may be compatible with biochemical consequences of thyroid autoimmunity that precede or accompany functional thyroid impairment. However, the lack of uniform separation between AIT and corresponding non-AIT phenotypes prevents this finding from being interpreted as evidence of an independent autoimmune effect.

This cautious interpretation is particularly important because autoimmune thyroid disease involves interactions among immune activation, thyroid-cell injury, environmental influences, and progressive functional changes rather than a single isolated biochemical pathway. Contemporary reviews describe Hashimoto thyroiditis as a multifactorial immune-mediated disorder involving interacting cellular, molecular, genetic, and environmental mechanisms [52,53]. The present plasma purine findings may therefore represent one component of a broader metabolic phenotype, but they should not be considered specific biomarkers of autoimmunity without independent validation.

A substantially different pattern emerged in erythrocytes, where purine-metabolite signals were broadly elevated across thyroid-disease groups relative to healthy controls, with limited separation among the clinical phenotypes. This divergence from the plasma profile is biologically plausible because erythrocytes are metabolically active cells whose glycolytic, pentose-phosphate, nucleotide-salvage, membrane-transport, and redox pathways can influence intracellular purine pools independently of circulating levels [54]. Their continuous exposure to oxygen and hemoglobin auto-oxidation further links intracellular metabolism to redox adaptation [55,56]. Thus, plasma and erythrocyte purine measurements should be regarded as complementary rather than interchangeable, while the particularly pronounced erythrocyte lipid-peroxidation signal in hypothyroidism without AIT may reflect additional impairment of red-cell redox stability.

The participant-level ratio analysis further showed that X/HX, UA/X, and UA/HX capture different aspects of the purine-metabolite profile rather than behaving as interchangeable indices. Plasma X/HX and UA/HX displayed clear between-group structure, whereas plasma UA/X was comparatively stable; in erythrocytes, the main phenotype-related difference involved X/HX. Together with the distinct distributions of individual purine metabolites, these findings support compartment-specific remodeling of the purine pool. Because these ratios are derived from relative spectrophotometric metabolite signals, they should be interpreted as exploratory indices of the relative distribution of sequential purine metabolites within each biological compartment, rather than as measures of XOR, XO, or XDH activity or of pathway flux. Biologically, these ratios may reflect the relative balance among sequential purine-metabolite pools, which can be influenced not only by terminal purine catabolism but also by nucleotide salvage, cellular release and uptake, membrane transport, and compartment-specific substrate availability. In erythrocytes, additional influences may include intracellular nucleotide turnover, erythrocyte age and turnover, and redox-dependent metabolic adaptation [54,56]. Thus, changes in X/HX, UA/X, or UA/HX may arise from several concurrent processes and should not be attributed to a single enzymatic mechanism. At the systemic level, measured plasma XOR activity has also been associated with cardiovascular risk in population-based studies, supporting the broader clinical relevance of purine–redox interactions while remaining distinct from the indirect spectrophotometric indices evaluated here [57].

One of the principal findings of the present study is the clear divergence between circulating and erythrocyte lipid-peroxidation profiles. Plasma TBARS-derived MDA was significantly elevated in all four thyroid-disease groups relative to healthy controls, indicating that increased circulating lipid peroxidation represents a broadly shared feature across the investigated thyroid phenotypes. In contrast, erythrocyte MDA showed a markedly more selective distribution, with the highest values in hypothyroidism without AIT and no corresponding elevation relative to controls in the other three thyroid-disease groups. This compartment-specific divergence is biologically important because it suggests that systemic oxidative remodeling and erythrocyte redox disturbance are not interchangeable phenomena. The generalized plasma pattern is consistent with previous evidence linking both hypothyroidism and autoimmune thyroid disease to systemic oxidative imbalance [58,59], whereas the selective erythrocyte response identifies a distinct cellular dimension of redox remodeling that would not be apparent from plasma measurements alone.

The absence of a clear plasma MDA separation among the clinical phenotypes also argues against a specifically autoimmune origin of the circulating oxidative-stress signal. Autoimmune thyroid disease involves closely interconnected immune, inflammatory, metabolic, and redox mechanisms, and these processes may additionally be modified by environmental and nutritional factors [60]. In the context of the present findings, the comparable plasma MDA signal across autoimmune and non-autoimmune phenotypes is therefore more consistent with convergence on partially shared systemic redox pathways than with a circulating oxidative marker specific to thyroid autoimmunity.

Erythrocyte MDA, however, showed a fundamentally different phenotype distribution. The selective elevation observed in hypothyroidism without AIT suggests that erythrocyte lipid peroxidation cannot be regarded simply as a cellular equivalent of the circulating plasma MDA pool. This difference is consistent with the specialized redox biology of erythrocytes, which are particularly susceptible to oxidative injury because of continuous oxygen exposure, hemoglobin auto-oxidation, and the presence of oxidizable membrane components [61]. Thus, the erythrocyte MDA pattern may reflect a phenotype-specific disturbance in red-cell redox homeostasis rather than merely greater systemic exposure to lipid-peroxidation products.

The prominence of erythrocyte lipid peroxidation in hypothyroidism without AIT is also compatible with the broader possibility that thyroid hormone deficiency itself can contribute to redox disturbance independently of autoimmune inflammation. In experimental non-autoimmune hypothyroidism, oxidative injury has been demonstrated in cardiovascular tissues, including increased MDA and altered antioxidant defenses [62]. These preclinical findings do not establish the mechanism responsible for the erythrocyte MDA changes observed in the present human study, but they support the biological plausibility that thyroid hormone deficiency itself may contribute to oxidative injury apart from autoimmune inflammation.

Taken together, these findings indicate that plasma and erythrocyte TBARS-derived MDA capture distinct aspects of oxidative biology rather than interchangeable measures of a single redox process [63,64].

The exploratory correlation analysis provides additional support for a phenotype- and compartment-dependent relationship between purine-metabolite profiles and lipid peroxidation. In the pooled cohort, the strongest associations involved plasma uric-acid-containing ratios, particularly UA/X in relation to plasma MDA. However, the group-stratified analyses demonstrated that these relationships were not uniform across thyroid phenotypes. The positive association between plasma UA/X and plasma MDA was reproduced within hypothyroidism without AIT, AIT with hypothyroidism, and euthyroid AIT, but was absent in nontoxic nodular goiter with euthyroidism and healthy controls. In contrast, the plasma UA/HX–plasma MDA relationship showed marked phenotype-dependent heterogeneity, being inverse in hypothyroidism without AIT, positive in AIT with hypothyroidism and euthyroid AIT, and absent in nontoxic nodular goiter. Associations with erythrocyte MDA were less consistent across individual phenotypes. These findings indicate that the pooled correlations cannot be interpreted as a single uniform biological relationship across the entire cohort and instead suggest phenotype-specific differences in the coupling between the relative plasma purine profile and oxidative status.

Importantly, these correlations were not adjusted for age, sex, BMI, eGFR, L-T4 exposure, or other metabolic and clinical covariates; therefore, residual confounding may have contributed to the magnitude and direction of the observed associations.

This interpretation is compatible with epidemiological evidence linking circulating uric acid to thyroid hormone status and thyroid hormone sensitivity, with such relationships varying according to sex, obesity, and other metabolic characteristics [65,66].

XOR provides a biologically plausible mechanistic link between terminal purine catabolism and oxidative stress because its interconvertible forms differ in their electron acceptors and capacity for ROS generation [67]. Measured plasma XOR activity has also been investigated as a circulating indicator of oxidative stress in clinical populations [68]. However, this general biological relationship should be distinguished from the findings of the present study. The spectrophotometrically determined purine-metabolite signals and the calculated X/HX, UA/X, and UA/HX ratios are exploratory indices of relative metabolite distribution and do not measure XOR, XO, or XDH activity. Although direct human studies have associated measured plasma XOR activity with vascular dysfunction independently of serum uric acid concentrations [69], the correlations observed in the present study cannot be used to infer XOR activation or XOR-derived ROS generation. Direct enzyme measurements would be required to test this mechanism.

Levothyroxine exposure should also be considered when interpreting the biochemical heterogeneity observed among participants. Correction of circulating thyroid hormone concentrations does not necessarily normalize every downstream redox or metabolic alteration. In a clinical comparison of women with autoimmune and non-autoimmune hypothyroidism rendered euthyroid by levothyroxine replacement, thiol–disulfide homeostasis remained shifted toward an oxidative state in both patient groups relative to healthy controls [70]. Consequently, treatment may correct thyroid hormone concentrations while leaving residual redox abnormalities, and levothyroxine exposure should therefore be regarded as a potential source of interindividual biochemical variability in the present cross-sectional analysis.

In the present cohort, L-T4 exposure was common in both hypothyroid groups, being recorded in 71.0% of participants with hypothyroidism without AIT and 80.0% of participants in the AIT-with-hypothyroidism group. Median weight-adjusted doses and treatment durations were also numerically similar between the two hypothyroid groups. Nevertheless, the erythrocyte MDA profiles differed markedly, whereas plasma MDA was elevated across all thyroid-disease groups. These findings suggest that L-T4 exposure alone is unlikely to account for the observed compartment-specific oxidative pattern; however, because treatment was not randomized and the study was cross-sectional, an independent effect of L-T4 treatment status, dose, or duration on oxidative parameters cannot be established. A separate treatment-stratified analysis was not undertaken because the untreated subgroups within the hypothyroid phenotypes were small and clinically selected, making post hoc treated-versus-untreated comparisons vulnerable to unstable estimates and confounding by indication. Accordingly, levothyroxine exposure was considered a potential source of variability rather than an independently tested determinant of the observed biochemical patterns.

From a translational perspective, the combined assessment of purine-metabolite profiles and lipid-peroxidation markers in both plasma and erythrocytes may provide information complementary to conventional thyroid evaluation based on TSH, fT4, thyroid autoantibodies, and structural assessment. Conventional diagnostic markers define thyroid functional and autoimmune status, whereas the biochemical parameters examined here capture additional dimensions of metabolic and redox remodeling. The observed compartment-specific patterns therefore raise the possibility that multi-parameter purine–redox profiling could contribute to metabolic phenotyping of patients who share similar conventional thyroid classifications but differ in their systemic or cellular biochemical response. Such profiling may also be of interest for longitudinal assessment during levothyroxine therapy, particularly for determining whether normalization of thyroid hormone concentrations is accompanied by normalization of purine and redox abnormalities. These potential applications require prospective validation, determination of analytical and clinical performance, and assessment of within-patient changes over time; the present cross-sectional study should therefore be regarded as a foundation for such translational investigation rather than as validation of a diagnostic or treatment-monitoring test.

Overall, the findings support phenotype- and compartment-dependent purine and lipid-peroxidation patterns rather than a uniform AIT-dependent metabolic shift. Future studies combining targeted quantitative metabolomics, direct XO/XDH activity assays, detailed renal and metabolic characterization, and longitudinal sampling will be required to resolve the mechanisms underlying these observations [71]. This direction is consistent with contemporary evidence linking thyroid dysfunction to oxidative stress, erythrocyte redox regulation, and purine-metabolism disturbances [72,73].

Nevertheless, the interpretation of these findings should take into account several methodological and design-related limitations of the present study.

First, this was a single-center cross-sectional study; therefore, the observed differences in purine-metabolite profiles, calculated relative indices, and lipid-peroxidation parameters represent associations at a single time point and do not establish causal or temporal relationships among thyroid dysfunction, autoimmune inflammation, purine metabolism, and oxidative stress. The single-center design and the predefined age range may also limit generalizability to broader patient populations.

Second, the purine-metabolite measurements and derived ratios should be interpreted within the analytical framework of the applied direct UV-spectrophotometric method. X/HX, UA/X, and UA/HX were calculated from relative extinction-based signals and represent dimensionless, method-specific exploratory indices of the relative distribution of sequential purine metabolites rather than molar product-to-substrate ratios, metabolic flux, or direct measures of XOR, XO, or XDH activity. In addition, fixed-wavelength spectrophotometry does not provide chromatographic separation; consequently, spectral overlap from endogenous UV-absorbing compounds and matrix-related effects cannot be fully excluded. Formal cross-validation against HPLC-UV or LC–MS/MS was not included in the original analytical protocol, and paired chromatographic measurements from the same study specimens were not available for retrospective method comparison. Future studies should therefore incorporate targeted chromatographic quantification and, where mechanistically relevant, direct XOR activity measurements.

Third, lipid peroxidation was assessed using the TBARS assay, and the results should therefore be interpreted as TBARS-derived MDA equivalents rather than as analytically specific measurements of MDA alone. The TBARS reaction may include contributions from other thiobarbituric-acid-reactive compounds, and some ex vivo oxidation during sample processing cannot be completely excluded. Although samples were analyzed in duplicate under standardized quality-control conditions, no exogenous antioxidant or metal-chelating agent was added during sample preparation, and formal matrix-specific intra-assay and inter-assay CVs were not separately established. These factors should be considered when interpreting the absolute analytical specificity of the lipid-peroxidation measurements.

Fourth, residual confounding cannot be excluded. The correlation analyses were exploratory and were based on unadjusted bivariate Spearman correlations; no multivariable covariate-adjusted modeling was prespecified or performed. Age, sex, BMI, eGFR, and L-T4 exposure were characterized and reported, but inflammatory markers such as hs-CRP, ESR, and NLR were not systematically available, and other potentially relevant factors, including dietary patterns, physical activity, lipid status, medications, and renal tubular handling of urate, were not comprehensively characterized. In addition, L-T4 treatment was clinically determined rather than randomized and was largely restricted to the hypothyroid phenotypes. Treatment-stratified analyses were not performed because the untreated subgroups were small, making post hoc treated-versus-untreated comparisons vulnerable to unstable estimates and confounding by indication. Accordingly, the present study cannot establish independent effects of L-T4 exposure or other clinical covariates on the observed purine-metabolite and redox patterns.

Finally, the study did not include longitudinal follow-up. It therefore remains uncertain which abnormalities in purine-metabolite distribution and lipid peroxidation are reversible after restoration or maintenance of euthyroidism and which may persist because of autoimmune, structural, cellular, or longer-term metabolic and redox mechanisms. Prospective longitudinal studies combining repeated sampling, targeted quantitative metabolomics, direct enzyme assessment, and more comprehensive clinical, renal, metabolic, treatment, and inflammatory characterization will be required to determine the temporal stability and mechanistic significance of the observed phenotype- and compartment-specific patterns.

## 5. Conclusions

The present study identified distinct plasma and erythrocyte patterns of purine-related parameters and lipid-peroxidation markers across the studied thyroid phenotypes. The observed between-group differences involved both uric acid and intermediate purine-related parameters, including guanine, hypoxanthine, adenine, and xanthine, as well as calculated purine-metabolite ratios.

In both AIT groups, plasma guanine and hypoxanthine were lower than in the corresponding non-AIT groups, whereas plasma X/HX and UA/HX were higher than in healthy controls. However, direct AIT-versus-non-AIT comparisons of these ratios within the same thyroid functional state were not significant. Therefore, these ratio findings indicate differences in the relative distribution of plasma purine metabolites but do not establish an independent autoimmune effect, altered metabolic flux, or XOR/XO/XDH enzymatic activity.

Plasma MDA was significantly elevated in all four thyroid-disease groups relative to controls, without significant differences among the clinical phenotypes. In contrast, erythrocyte MDA showed a more selective pattern, with the highest values in hypothyroidism without AIT and significant differences from controls and from the other three thyroid-disease groups. These findings demonstrate that plasma and erythrocyte lipid-peroxidation markers do not show parallel distributions across the studied thyroid phenotypes.

Taken together, the pooled and group-stratified correlation analyses indicate phenotype-dependent rather than uniform relationships between plasma uric-acid-containing ratios and lipid-peroxidation status. The plasma UA/X–plasma MDA association was reproduced in several thyroid-disease phenotypes, whereas the UA/HX–plasma MDA relationship showed substantial variation in both magnitude and direction across groups. These associations remain exploratory and do not establish causality or XOR-dependent ROS generation.

## Figures and Tables

**Figure 1:**
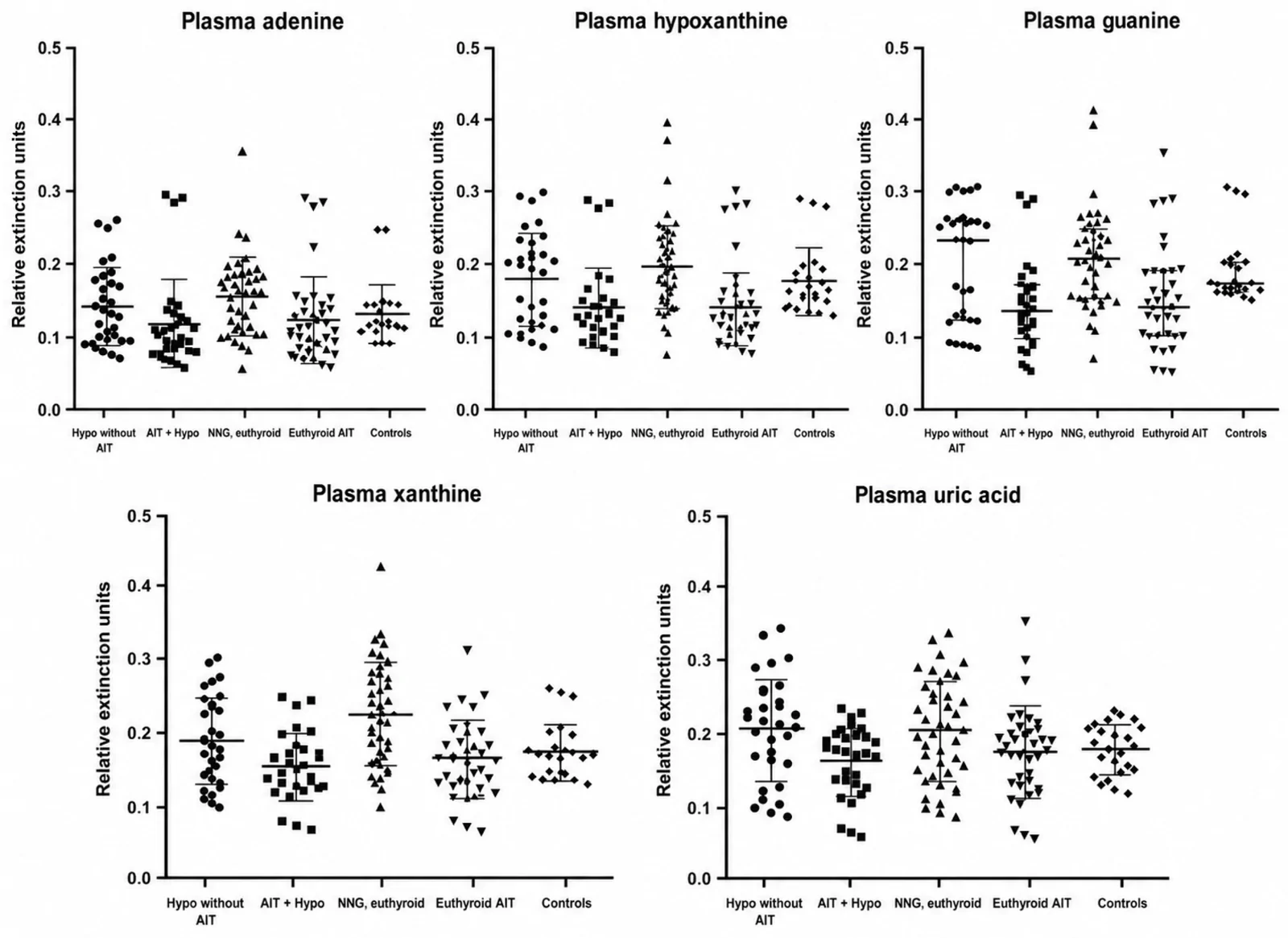
Participant-level distributions of plasma purine-metabolite parameters across the five study groups. Individual points represent participant-level measurements; horizontal lines indicate means and vertical error bars indicate standard deviations (SD). Plasma MDA is expressed as µmol/L, whereas erythrocyte MDA is normalized to hemoglobin and expressed as nmol/g Hb. Plasma purine-metabolite values are expressed in relative extinction units. Group labels are defined in Section 2.1 and Table 1.

**Figure 2:**
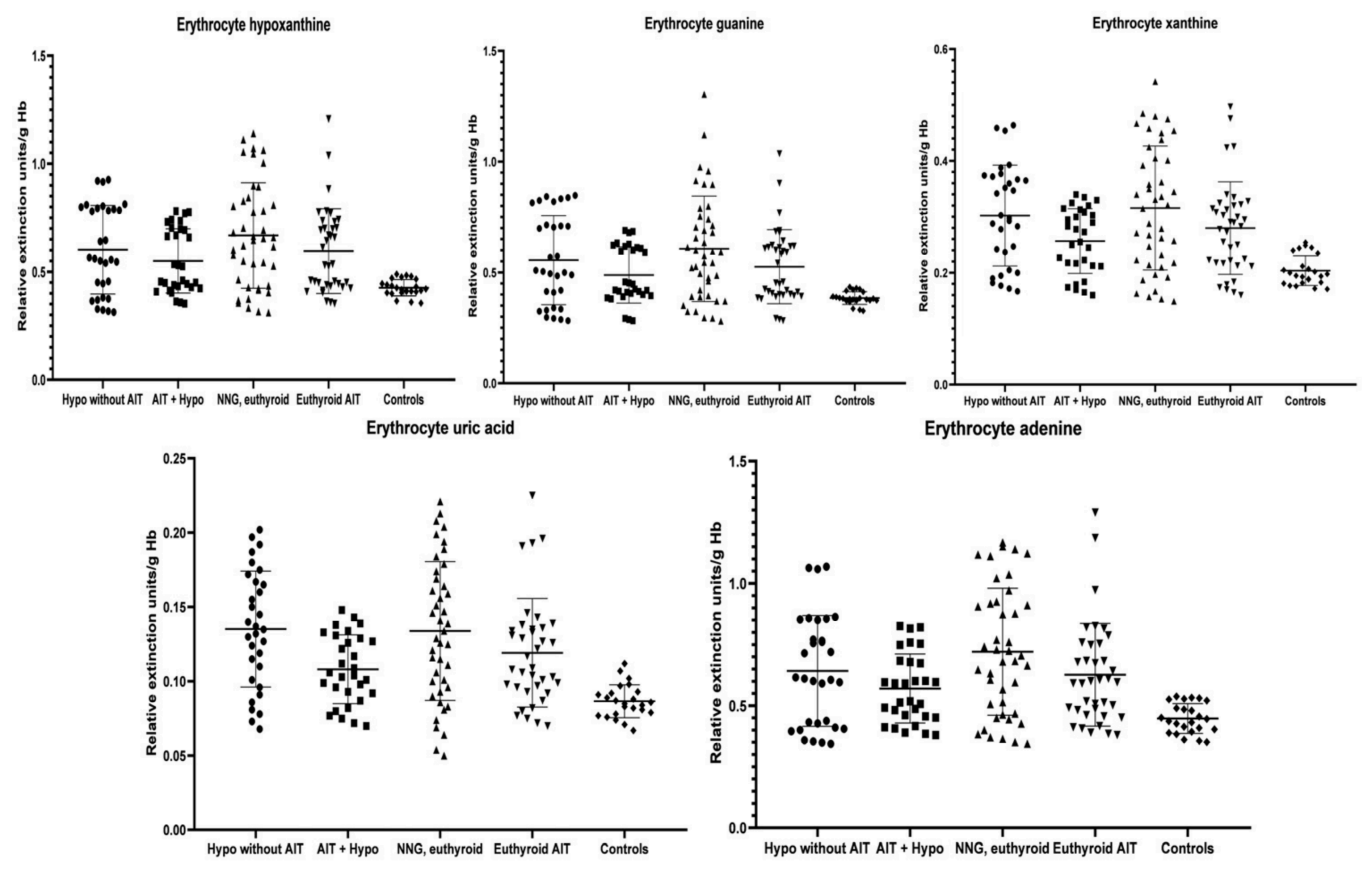
Participant-level distributions of erythrocyte purine-metabolite parameters across the five study groups. Individual points represent participant-level measurements; horizontal lines indicate means and vertical error bars indicate standard deviations (SD). Erythrocyte purine-metabolite values are normalized to hemoglobin and expressed as relative extinction units/g Hb. Group labels are defined in Section 2.1 and Table 1.

**Figure 3:**
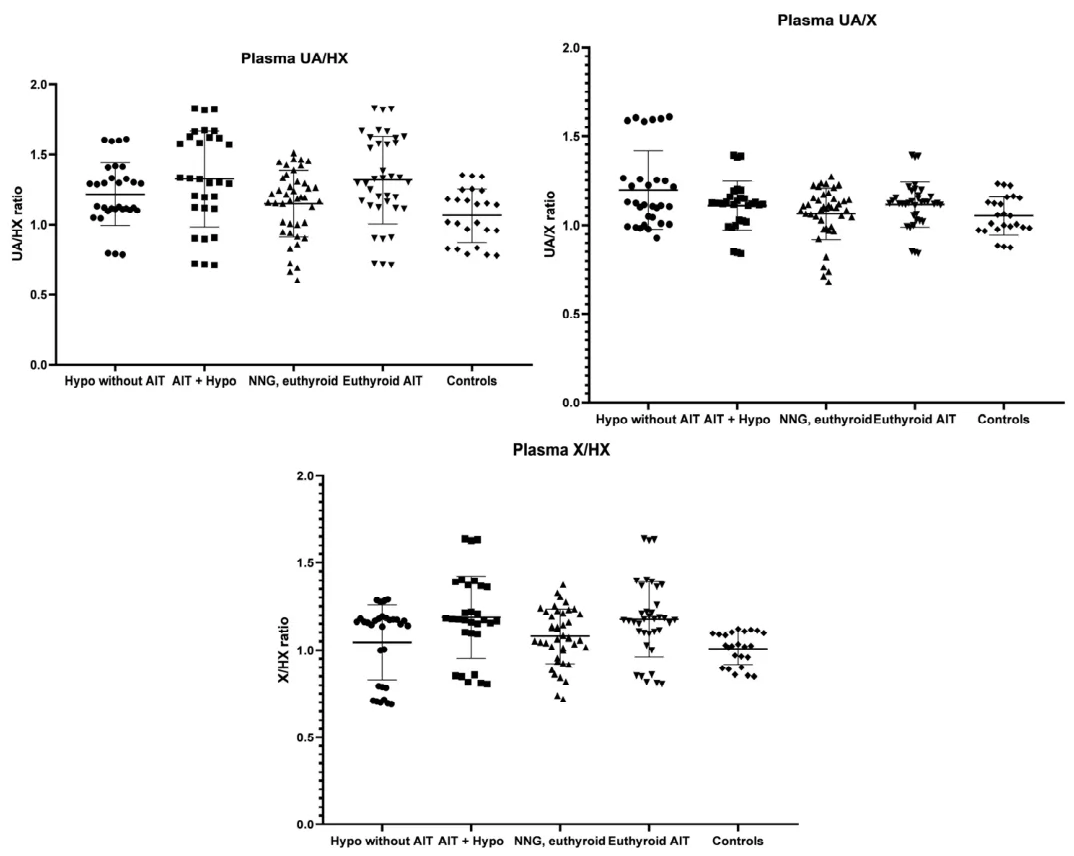
Participant-level distributions of plasma purine-metabolite ratios across the five study groups. Individual points represent participant-level measurements; horizontal lines indicate means and vertical error bars indicate standard deviations (SD). Ratios were calculated separately for each participant from the corresponding purine-metabolite measurements within plasma and are presented as dimensionless method-specific spectrophotometric indices. X, xanthine; HX, hypoxanthine; UA, uric acid. Group labels are defined in Section 2.1 and Table 1.

**Figure 4:**
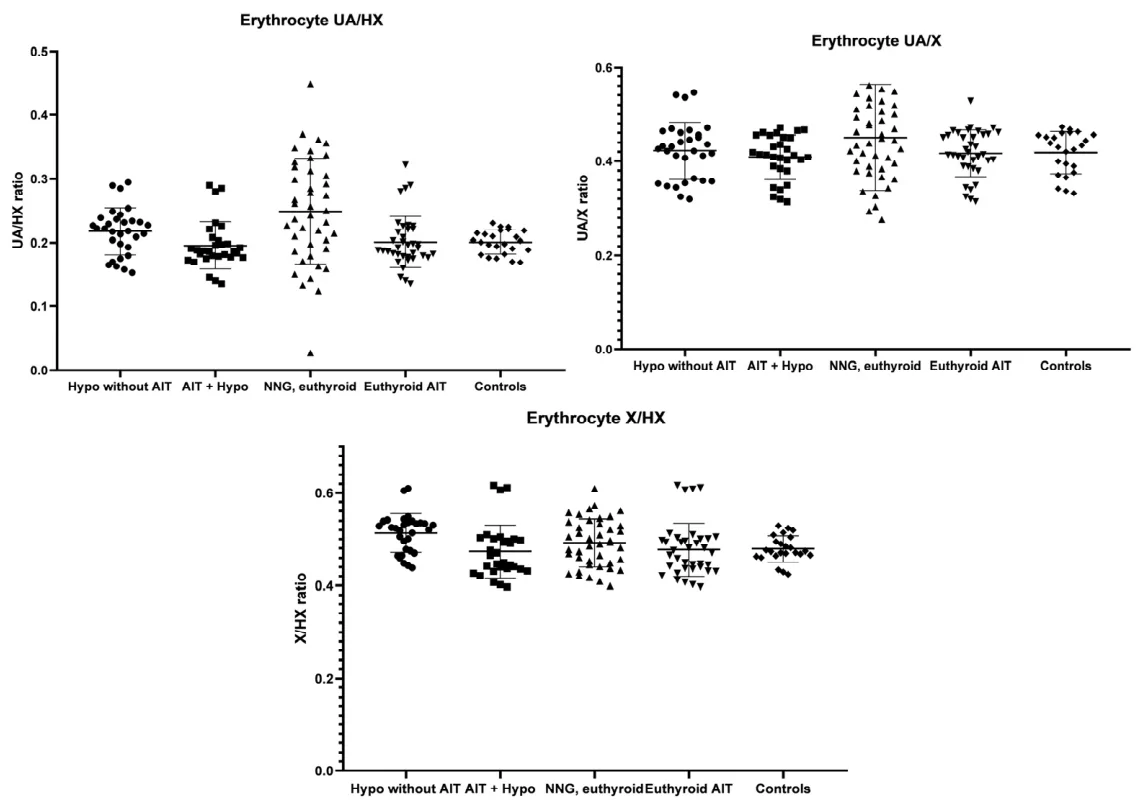
Participant-level distributions of erythrocyte purine-metabolite ratios across the five study groups. Individual points represent participant-level measurements; horizontal lines indicate means and vertical error bars indicate standard deviations (SD). Ratios were calculated separately for each participant from the corresponding purine-metabolite measurements within the erythrocyte compartment and are presented as dimensionless method-specific spectrophotometric indices. X, xanthine; HX, hypoxanthine; UA, uric acid. Group labels are defined in Section 2.1 and Table 1.

**Figure 5:**
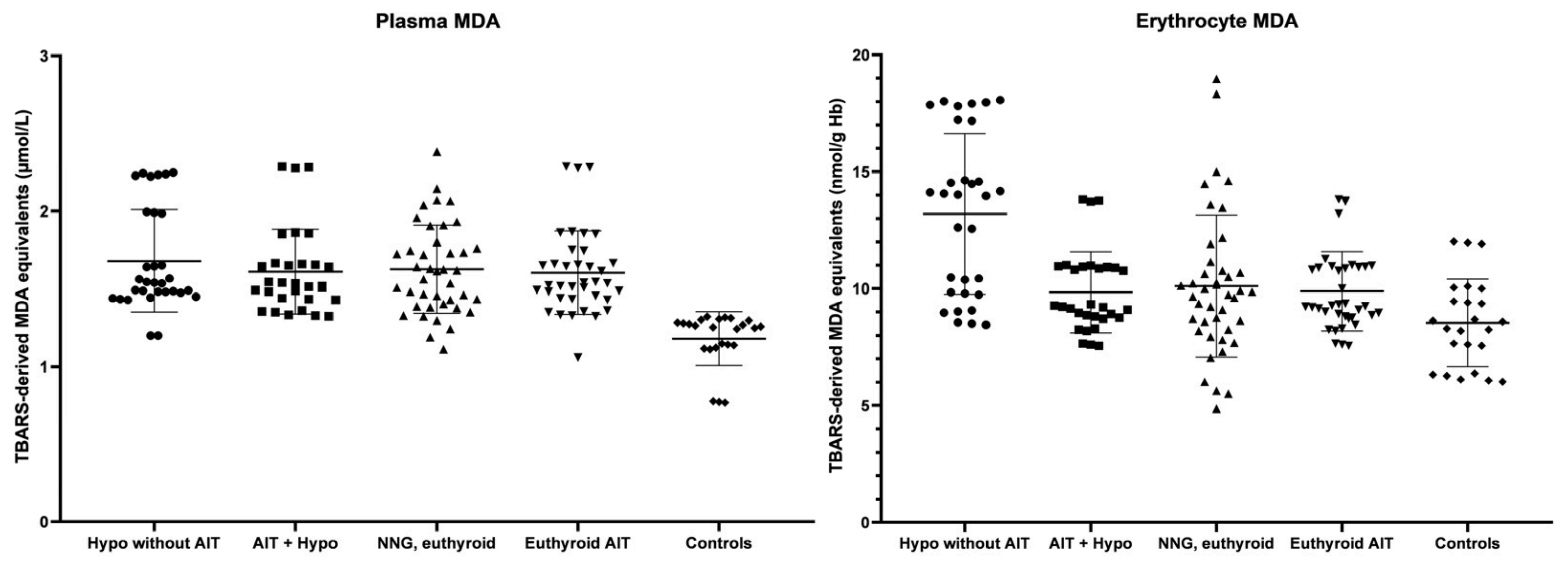
Participant-level distributions of TBARS-derived malondialdehyde equivalents in plasma and erythrocytes across the five study groups. Individual points represent participant-level measurements; horizontal lines indicate means and vertical error bars indicate standard deviations (SD). TBARS, thiobarbituric acid-reactive substances; MDA, malondialdehyde; Hb, hemoglobin. Group labels are defined in Section 2.1 and Table 1.

**Table 1:** Baseline demographic, anthropometric, thyroid-related, treatment, and renal characteristics of the study groups.

Characteristic	Hypo Without AIT (*n* = 31)	AIT + Hypo (*n* = 30)	NNG, Euthyroid (*n* = 42)	Euthyroid AIT (*n* = 37)	Controls (*n* = 24)
Age, years, mean ± SD	41.19 ± 7.97	41.03 ± 8.95	39.93 ± 8.50	43.65 ± 8.90	40.54 ± 8.66
Age, years, median (Q1–Q3)	41 (34–47)	42.5 (33.3–48.0)	39 (33–46)	44 (38–51)	40 (35.8–44.5)
Female, *n* (%)	25 (80.6%)	26 (86.7%)	32 (76.2%)	31 (83.8%)	16 (66.7%)
Male, *n* (%)	6 (19.4%)	4 (13.3%)	10 (23.8%)	6 (16.2%)	8 (33.3%)
BMI, kg/m^2^, median (Q1–Q3)	29.3 (27.1–31.8)	26.2 (24.7–29.1)	27.9 (25.3–29.3)	26.2 (24.8–29.0)	25.8 (23.5–27.0)
TSH, mIU/L, median (Q1–Q3)	7.95 (5.16–11.66)	8.52 (5.89–13.79)	2.19 (1.71–2.66)	2.00 (1.35–2.61)	1.85 (1.45–2.08)
fT4, pmol/L, median (Q1–Q3)	12.3 (8.6–13.7)	11.8 (8.5–13.6)	16.3 (14.4–17.2)	14.2 (13.2–15.9)	15.7 (15.1–17.0)
anti-TPO, IU/mL, median (Q1–Q3)	14.5 (8.7–20.0)	221.0 (162.7–415.3)	15.3 (9.8–18.7)	170.2 (81.0–369.4)	11.5 (8.3–16.2)
anti-TG, IU/mL, median (Q1–Q3)	41.3 (31.1–67.0)	213.9 (112.9–269.0)	36.1 (22.9–57.5)	218.6 (119.5–408.4)	36.2 (24.0–45.2)
Subclinical hypothyroidism, *n* (%)	20 (64.5%)	18 (60.0%)	—	—	—
Overt hypothyroidism, *n* (%)	11 (35.5%)	12 (40.0%)	—	—	—
eGFR, mL/min/1.73 m^2^, median (Q1–Q3)	91.4 (84.2–104.6)	92.5 (82.8–99.1)	101.1 (88.6–112.5)	96.9 (84.0–104.0)	100.5 (95.6–114.2)
L-T4 use, *n* (%)	22 (71.0%)	24 (80.0%)	0 (0.0%)	0 (0.0%)	0 (0.0%)
L-T4 dose, µg/kg/day, median (Q1–Q3)	1.00 (0.73–1.28)	0.91 (0.73–1.18)	—	—	—
L-T4 treatment duration, months, median (Q1–Q3)	39.5 (23.8–70.5)	46.5 (26.0–74.3)	—	—	—

Note: Continuous variables are presented as mean ± SD or median [Q1–Q3], as indicated; categorical variables are presented as *n* (%). L-T4 dose and treatment duration are presented for participants receiving levothyroxine only. A dash (—) indicates that the characteristic was not applicable. AIT, autoimmune thyroiditis; BMI, body mass index; TSH, thyroid-stimulating hormone; fT4, free thyroxine; anti-TPO, antibodies to thyroid peroxidase; anti-TG, antibodies to thyroglobulin; eGFR, estimated glomerular filtration rate; L-T4, levothyroxine. Group nomenclature used throughout the tables: Hypo without AIT, hypothyroidism without autoimmune thyroiditis; AIT + Hypo, autoimmune thyroiditis with hypothyroidism; NNG, euthyroid, nontoxic nodular goiter with euthyroidism; Euthyroid AIT, euthyroid autoimmune thyroiditis; Controls, healthy controls.

**Table 2:** Plasma levels of purine metabolites in patients with different thyroid-disease phenotypes.

Metabolite	Hypo Without AIT (*n* = 31)	AIT + Hypo (*n* = 30)	NNG, Euthyroid (*n* = 42)	Euthyroid AIT (*n* = 37)	Controls (*n* = 24)	H (df = 4)	*p* Value
Guanine	0.2260 (0.1230–0.2550)	0.1350 (0.1060–0.1630)	0.2090 (0.1578–0.2480)	0.1410 (0.1065–0.1930)	0.1685 (0.1618–0.2035)	29.66	<0.0001
Hypoxanthine	0.1810 (0.0980–0.2210)	0.1130 (0.0880–0.1380)	0.1880 (0.1363–0.2190)	0.1160 (0.0930–0.1660)	0.1455 (0.1320–0.1758)	28.78	<0.0001
Adenine	0.1280 (0.0770–0.1790)	0.0980 (0.0720–0.1230)	0.1635 (0.1118–0.1843)	0.1040 (0.0790–0.1400)	0.1200 (0.1105–0.1465)	23.04	0.0001
Xanthine	0.1250 (0.1250–0.2280)	0.1410 (0.1140–0.1670)	0.1805 (0.1475–0.2378)	0.1490 (0.1170–0.1910)	0.1590 (0.1285–0.1853)	16.84	0.0021
Uric acid	0.1980 (0.1560–0.2500)	0.1665 (0.1270–0.1860)	0.1910 (0.1510–0.2548)	0.1680 (0.1310–0.1940)	0.1720 (0.1320–0.1998)	13.56	0.0088

Note: Group labels are defined in Section 2.1 and Table 1. Data are presented as median [Q1–Q3]. Overall *p* values were calculated using the Kruskal–Wallis test. Purine-metabolite parameters were determined by direct UV spectrophotometry and are reported in relative extinction units.

**Table 3:** Levels of purine metabolites in the erythrocyte fraction of blood in patients with different thyroid-disease phenotypes.

Metabolite	Hypo Without AIT (*n* = 31)	AIT + Hypo (*n* = 30)	NNG, Euthyroid (*n* = 42)	Euthyroid AIT (*n* = 37)	Controls (*n* = 24)	H (df = 4)	*p* Value
Guanine	0.4850 (0.3250–0.7100)	0.4315 (0.3910–0.6090)	0.5845 (0.3943–0.7443)	0.4480 (0.3920–0.6130)	0.3740 (0.3683–0.4045)	30.59	<0.0001
Hypoxanthine	0.5410 (0.3650–0.7830)	0.4880 (0.4240–0.6930)	0.6510 (0.4340–0.8320)	0.5260 (0.4290–0.7155)	0.4145 (0.4000–0.4583)	26.13	<0.0001
Adenine	0.5910 (0.3960–0.8510)	0.5495 (0.4520–0.6740)	0.6945 (0.4670–0.9128)	0.5910 (0.4670–0.7490)	0.4365 (0.3890–0.5108)	28.84	<0.0001
Xanthine	0.2780 (0.1900–0.3670)	0.2590 (0.2120–0.3030)	0.3180 (0.2195–0.4100)	0.2730 (0.2125–0.3150)	0.1915 (0.1760–0.2268)	32.29	<0.0001
Uric acid	0.1240 (0.0810–0.1300)	0.0975 (0.0720–0.1260)	0.1290 (0.0953–0.1690)	0.1030 (0.0825–0.1310)	0.0800 (0.0748–0.0878)	32.55	<0.0001

Note: Group labels are defined in Section 2.1 and Table 1. Data are presented as median [Q1–Q3]. Overall *p* values were calculated using the Kruskal–Wallis test. Erythrocyte purine-metabolite parameters were determined by direct UV spectrophotometry, normalized to hemoglobin, and are reported as relative extinction units/g Hb. Hb, hemoglobin.

**Table 4:** Relative purine-metabolite indices in plasma and erythrocytes in patients with different thyroid-disease phenotypes.

Matrix	Ratio	Hypo Without AIT (*n* = 31)	AIT + Hypo (*n* = 30)	NNG, Euthyroid (*n* = 42)	Euthyroid AIT (*n* = 37)	Controls (*n* = 24)	H (df = 4)	*p* Value
Plasma	X/HX	1.158 (0.7849–1.175)	1.170 (1.092–1.364)	1.064 (0.9887–1.214)	1.167 (1.092–1.311)	1.020 (0.9083–1.094)	22.84	0.0001
Plasma	UA/X	1.105 (1.000–1.248)	1.117 (1.019–1.150)	1.096 (0.9914–1.150)	1.120 (1.037–1.159)	1.026 (0.9721–1.145)	9.064	0.0595
Plasma	UA/HX	1.110 (1.094–1.324)	1.306 (1.112–1.614)	1.190 (0.9871–1.312)	1.289 (1.123–1.591)	1.075 (0.8572–1.225)	19.08	0.0008
Erythrocytes	X/HX	0.5139 (0.4651–0.5314)	0.4529 (0.4309–0.4929)	0.4670 (0.4391–0.5073)	0.4658 (0.4309–0.4932)	0.4678 (0.4614–0.4993)	19.95	0.0005
Erythrocytes	UA/X	0.4119 (0.3542–0.4460)	0.4063 (0.3796–0.4500)	0.3938 (0.3668–0.4738)	0.4092 (0.3840–0.4507)	0.4271 (0.3721–0.4510)	1.119	0.8913
Erythrocytes	UA/HX	0.2173 (0.1934–0.2292)	0.1781 (0.1725–0.1983)	0.1782 (0.1660–0.2407)	0.1787 (0.1725–0.2209)	0.1945 (0.1748–0.2122)	7.853	0.0971

Note: Data are presented as median [Q1–Q3]. Ratios were calculated at the individual-participant level from the corresponding relative spectrophotometric signals and are presented as dimensionless method-specific indices. X, xanthine; HX, hypoxanthine; UA, uric acid. Overall *p*-values were calculated using the Kruskal–Wallis test. Group labels are defined in Section 2.1 and Table 1.

**Table 5:** TBARS-derived MDA equivalents in plasma and erythrocytes across the study groups.

Matrix	Hypo Without AIT (*n* = 31)	AIT + Hypo (*n* = 30)	NNG, Euthyroid (*n* = 42)	Euthyroid AIT (*n* = 37)	Controls (*n* = 24)	H (df = 4)	*p* Value
Plasma MDA, μmol/L	1.535 (1.429–1.986)	1.522 (1.429–1.641)	1.615 (1.396–1.800)	1.535 (1.429–1.694)	1.244 (1.118–1.283)	59.87	<0.0001
Erythrocyte MDA, nmol/g Hb	13.97 (9.744–17.18)	9.167 (8.718–10.77)	9.808 (8.205–10.87)	9.231 (8.718–10.77)	8.397 (6.603–9.840)	33.82	<0.0001

Note: Data are presented as median [Q1–Q3]. Plasma MDA is expressed in µmol/L, and erythrocyte MDA is normalized to hemoglobin and expressed in nmol/g Hb. Overall *p* values were calculated using the Kruskal–Wallis test. Group labels are defined in Section 2.1 and Table 1. MDA, malondialdehyde; Hb, hemoglobin.

**Table 6:** Spearman correlations between purine-metabolite ratios and TBARS-derived MDA equivalents in plasma and erythrocytes in the full analytic cohort (*n* = 164).

Purine-Metabolite Ratio	MDA Variable	Spearman ρ	95% CI	*p* Value
Plasma X/HX	Plasma MDA	0.089	−0.066 to 0.239	0.2593
Plasma X/HX	Erythrocyte MDA	0.038	−0.115 to 0.191	0.6246
Plasma UA/HX	Plasma MDA	0.228	0.078 to 0.369	0.0033
Plasma UA/HX	Erythrocyte MDA	0.134	−0.020 to 0.281	0.0873
Plasma UA/X	Plasma MDA	0.376	0.237 to 0.501	6.86 × 10^−7^
Plasma UA/X	Erythrocyte MDA	0.210	0.059 to 0.352	0.0068
Erythrocyte X/HX	Plasma MDA	−0.162	−0.307 to −0.009	0.0387
Erythrocyte X/HX	Erythrocyte MDA	0.102	−0.052 to 0.251	0.1933
Erythrocyte UA/HX	Plasma MDA	−0.121	−0.270 to 0.032	0.1215
Erythrocyte UA/HX	Erythrocyte MDA	0.035	−0.119 to 0.187	0.6560
Erythrocyte UA/X	Plasma MDA	−0.024	−0.176 to 0.130	0.7638
Erythrocyte UA/X	Erythrocyte MDA	−0.052	−0.204 to 0.102	0.5050

Note: X, xanthine; HX, hypoxanthine; UA, uric acid; MDA, malondialdehyde; CI, confidence interval. Values are presented as Spearman’s rank correlation coefficient (ρ), 95% CI, and two-sided *p* value. Correlations are unadjusted bivariate associations calculated from participant-level observations in the full analytic cohort (*n* = 164).

**Table 7:** Group-stratified Spearman correlations of plasma UA/HX and UA/X with plasma and erythrocyte TBARS-derived MDA equivalents.

Clinical Group	UA/HX vs. Plasma MDA, ρ (*p*)	UA/HX vs. Erythrocyte MDA, ρ (*p*)	UA/X vs. Plasma MDA, ρ (*p*)	UA/X vs. Erythrocyte MDA, ρ (*p*)
Hypothyroidism without AIT	−0.599 (0.0004)	−0.279 (0.1290)	0.418 (0.0194)	0.375 (0.0374)
AIT with hypothyroidism	0.552 (0.0016)	0.195 (0.3015)	0.867 (<0.0001)	0.079 (0.6771)
Nontoxic nodular goiter with euthyroidism	−0.004 (0.9785)	−0.034 (0.8325)	0.053 (0.7399)	0.046 (0.7706)
Euthyroid AIT	0.494 (0.0019)	0.184 (0.2758)	0.687 (<0.0001)	0.052 (0.7591)
Healthy controls	−0.390 (0.0593)	0.214 (0.3147)	−0.146 (0.4949)	0.405 (0.0498)

Note: AIT, autoimmune thyroiditis; UA, uric acid; HX, hypoxanthine; X, xanthine; MDA, malondialdehyde. Values are presented as Spearman’s rank correlation coefficient (ρ), with two-sided *p*-values in parentheses. Group-stratified analyses were exploratory, and *p*-values were not adjusted for multiple subgroup comparisons.

## Data Availability

The data presented in this study are available from the corresponding author upon reasonable request. The data are not publicly available due to privacy and ethical restrictions.

## Abbreviations

AIT	Autoimmune thyroiditis
anti-TG	Antibodies to thyroglobulin
anti-TPO	Antibodies to thyroid peroxidase
ATP	Adenosine triphosphate
BMI	Body mass index
CI	Confidence interval
CKD-EPI	Chronic Kidney Disease Epidemiology Collaboration
CV	Coefficient of variation
eGFR	Estimated glomerular filtration rate
ELISA	Enzyme-linked immunosorbent assay
ESR	Erythrocyte sedimentation rate
fT3	Free triiodothyronine
fT4	Free thyroxine
H_2_O_2_	Hydrogen peroxide
Hb	Hemoglobin
HPLC-UV	High-performance liquid chromatography with ultraviolet detection
hs-CRP	High-sensitivity C-reactive protein
HX	Hypoxanthine
LC–MS/MS	Liquid chromatography–tandem mass spectrometry
L-T4	Levothyroxine
MDA	Malondialdehyde
NAD^+^	Nicotinamide adenine dinucleotide
NLR	Neutrophil-to-lymphocyte ratio
NNG	Nontoxic nodular goiter
ROS	Reactive oxygen species
SD	Standard deviation
TBA	Thiobarbituric acid
TBARS	Thiobarbituric acid-reactive substances
TCA	Trichloroacetic acid
TG	Thyroglobulin
TMP	1,1,3,3-Tetramethoxypropane
TPO	Thyroid peroxidase
TSH	Thyroid-stimulating hormone
UA	Uric acid
X	Xanthine
XDH	Xanthine dehydrogenase
XO	Xanthine oxidase
XOR	Xanthine oxidoreductase
